# Satellite Ocean Colour: Current Status and Future Perspective

**DOI:** 10.3389/fmars.2019.00485

**Published:** 2019-08-29

**Authors:** Steve Groom, Shubha Sathyendranath, Yai Ban, Stewart Bernard, Robert Brewin, Vanda Brotas, Carsten Brockmann, Prakash Chauhan, Jong-kuk Choi, Andrei Chuprin, Stefano Ciavatta, Paolo Cipollini, Craig Donlon, Bryan Franz, Xianqiang He, Takafumi Hirata, Tom Jackson, Milton Kampel, Hajo Krasemann, Samantha Lavender, Silvia Pardo-Martinez, Frédéric Mélin, Trevor Platt, Rosalia Santoleri, Jozef Skakala, Blake Schaeffer, Marie Smith, Francois Steinmetz, Andre Valente, Menghua Wang

**Affiliations:** 1 Plymouth Marine Laboratory, Plymouth, United Kingdom; 2 National Centre for Earth Observation, Plymouth Marine Laboratory, Plymouth, United Kingdom; 3 State Key Laboratory of Satellite Ocean, Environment Dynamics, Second Institute of Oceanography, Ministry of Natural Resources, Hangzhou, China; 4 CSIR Earth Systems Earth Observation, CSIR – NRE, Cape Town, South Africa; 5 MARE, Faculdade de Ciências, Universidade de Lisboa, Lisbon, Portugal; 6 Brockmann Consult, Geesthacht, Germany; 7 Indian Institute of Remote Sensing, Dehradun, India; 8 KIOST-PML Science Lab, Korea Institute of Ocean Science and Technology, Plymouth, United Kingdom; 9 Telespazio VEGA UK Ltd. for ESA Climate Office, European Centre for Space Applications and Telecommunications, European Space Agency, Didcot, United Kingdom; 10 European Space Research and Technology Centre, European Space Agency, Noordwijk, Netherlands; 11 Goddard Space Flight Center, NASA, Greenbelt, MD, United States; 12 Arctic Research Center, Hokkaido University, Sapporo, Japan; 13 Instituto Nacional de Pesquisas Espaciais São Jose dos Campos, São Paulo, Brazil; 14 Helmholtz-Zentrum Geesthacht – Zentrum für Materialund Küstenforschung GmbH, Geesthacht, Germany; 15 Pixalytics Ltd., Plymouth, United Kingdom; 16 European Commission, Joint Research Centre (JRC), Ispra, Italy; 17 Consiglio Nazionale delle Ricerche, Rome, Italy; 18 Office of Research and Development, United States Environmental Protection Agency, Research Triangle, NC, United States; 19 HYGEOS, Lille, France; 20 Marine Ecosystems and Climate Branch, NOAA NESDIS STAR, College Park, MD, United States

**Keywords:** ocean colour, phytoplankton, ground-segment, climate data records, water-quality, capacity building

## Abstract

Spectrally resolved water-leaving radiances (ocean colour) and inferred chlorophyll concentration are key to studying phytoplankton dynamics at seasonal and interannual scales, for a better understanding of the role of phytoplankton in marine biogeochemistry; the global carbon cycle; and the response of marine ecosystems to climate variability, change and feedback processes. Ocean colour data also have a critical role in operational observation systems monitoring coastal eutrophication, harmful algal blooms, and sediment plumes. The contiguous ocean-colour record reached 21 years in 2018; however, it is comprised of a number of one-off missions such that creating a consistent time-series of ocean-colour data requires merging of the individual sensors (including MERIS, Aqua-MODIS, SeaWiFS, VIIRS, and OLCI) with differing sensor characteristics, without introducing artefacts. By contrast, the next decade will see consistent observations from operational ocean colour series with sensors of similar design and with a replacement strategy. Also, by 2029 the record will start to be of sufficient duration to discriminate climate change impacts from natural variability, at least in some regions. This paper describes the current status and future prospects in the field of ocean colour focusing on large to medium resolution observations of oceans and coastal seas. It reviews the user requirements in terms of products and uncertainty characteristics and then describes features of current and future satellite ocean-colour sensors, both operational and innovative. The key role of *in situ* validation and calibration is highlighted as are ground segments that process the data received from the ocean-colour sensors and deliver analysis-ready products to end-users. Example applications of the ocean-colour data are presented, focusing on the climate data record and operational applications including water quality and assimilation into numerical models. Current capacity building and training activities pertinent to ocean colour are described and finally a summary of future perspectives is provided.

## INTRODUCTION

Satellite observation of ocean-colour radiometry involves detection of spectral variations in the water-leaving radiance (or reflectance), which is the sunlight backscattered out of the ocean after interaction with water and its constituents. In the open ocean the signal is primarily dependent on phytoplankton which contain photosynthetic pigments, primarily chlorophyll-a (chl-a) and an assemblage of other pigments, and which coexist together with associated detrital and coloured dissolved organic matter (CDOM) that are related to the phytoplankton. Coastal waters are more complex optically on account of the additional influences of re-suspended particulates, or river run-off which could contain terrestrial suspended particulates or CDOM which are independent of the phytoplankton assemblage (International Ocean Colour Coordinating Group [IOCCG], 2000).

To infer the concentration of chl-a or other optically active constituents from ocean-colour data, algorithms have been constructed relating characteristics of the water signal to the property of interest. These may be empirical algorithms (e.g., O’Reilly et al., 1998) or more complex semi-analytical approaches (e.g., Lee et al., 2002). However, because of the addition of light scattered by the atmosphere into the satellite view, the ocean colour signal is a relatively small part (<10%) of the satellite-detected top-of-atmosphere (TOA) radiance; so highly accurate atmospheric correction schemes are needed to retrieve the water signal (Wang et al., 2009). Furthermore, because the water signal is needed to within 5% (GCOS, 2016), the spaceborne sensor needs to retrieve TOA signal to 0.5% (International Ocean Colour Coordinating Group [IOCCG], 2012) and, hence, be well calibrated to avoid residual errors propagating to the water signal. This is accomplished through a combination of preflight calibration, post-launch onboard calibration of observation of external targets (such as the sun, moon or on the Earth) and a ‘system vicarious calibration’ undertaken to compare the expected TOA signal based on the water signal and atmospheric components with that measured by the sensor (see section ‘Ocean Colour EO Sensors and in situ Observations’). The system vicarious calibration is considered essential to reach the 0.5% requirement (International Ocean Colour Coordinating Group [IOCCG], 2012).

The first ocean colour sensor was the proof-of-concept Coastal Zone Color Scanner (CZCS: Gordon et al., 1980; Hovis et al., 1980) launched in 1978 and operated until 1986. After this there was a gap in the record until the launch of the Japanese Ocean Color Temperature Scanner (OCTS) in 1996 and the United States Sea-viewing Wide field-of-view Sensor (SeaWiFS) in 1997, which marked the start of the contiguous ocean colour era. Since then there have been further missions, typically of one-off instruments. This situation is, however, changing with the recent launches of operational ocean-colour sensor series (NOAA JPSS VIIRS) and ESA Sentinel 3 OLCI (see section ‘Ocean Colour EO Sensors and in situ Observations’).

The International Ocean Colour Coordinating Group (IOCCG^1^) has been, and continues to be, the principal forum for space agencies, domain experts and user representatives to discuss coordination and integration of the ocean-colour field. Key activities include formation of scientific working groups and the resulting reports and protocols that describe requirements and developments in the field. Importantly, these reports are freely available and so accessible to scientists in developing countries who may not have journal access. IOCCG report 8 ‘Why ocean colour’ (International Ocean Colour Coordinating Group [IOCCG], 2008) gave an overview of the varied applications of ocean colour for academic and societal applications, while other reports have focused more on specific issues including, *inter alia*: mission requirements for sensors (International Ocean Colour Coordinating Group [IOCCG], 2012), atmospheric correction (International Ocean Colour Coordinating Group [IOCCG], 2010), fisheries applications (International Ocean Colour Coordinating Group [IOCCG], 2009), phytoplankton functional types (International Ocean Colour Coordinating Group [IOCCG], 2014), and the most recent in the series focused on inland and coastal water quality (International Ocean Colour Coordinating Group [IOCCG], 2018). IOCCG initiated the biennial International Ocean Colour Coordinating Group [IOCCG], 2013 with an aim of bringing together the ocean colour community; the fourth meeting was in Korea in 2019.

This review starts with a summary of the user community requirements, then discusses current and upcoming satellite EO missions and *in situ* data, including citizen science/crowdsourcing, that support the ocean-colour sensors. It then describes ground segments, which take the raw ocean-colour data to generate intermediary or end products for users and provide data delivery and access mechanisms. Next, it discusses climate data records and presents example operational applications, considering how current and future developments in EO are likely to make an impact. The review discusses ongoing activities focused on ocean-colour relevant capacity building and training. Finally, a summary is given on perspectives for ocean colour in integrated, sustained observations for ocean, shelf and off-shore coastal applications but not on near-coastal, estuarine or inland water observation using high-resolution sensors. Furthermore, it does not cover polarimetry or LiDAR observations that are both described in Jamet et al. (2019).

## USER REQUIREMENTS

In considering user requirements it is instructive to review how community white papers from OceanObs’09 articulated the requirements for ocean-colour observations from a variety of perspectives and how these have led to progress in the subsequent decade. Bonekamp et al. (2010) discussed how the operational requirements of satellite observations in general, including ocean colour, differed from research requirements, and how science-driven single missions were transitioning to multi-mission, sustained ocean observations to meet a variety of operational requirements. The paper by Yoder et al. (2010) led to the development of an ocean-colour radiometry virtual constellation (OCR-VC) by the Committee on Earth Observation Satellites (CEOS^2^) to ensure sustainability of ocean colour observations through the coordinated efforts of the individual national space agencies. Yoder et al. (2010) also identified a number of operational applications for ocean colour, for health (identification of pollution, eutrophication, and harmful algal blooms; impacts of aerosols on health); climate (contribution to carbon observations in the oceans); marine ecosystems; fisheries; and aquaculture. They showed how the ocean-colour capabilities were beginning to meet the requirements identified by the various tasks of the Group on Earth Observations (GEO). The community white paper by Forget et al. (2010) presented the ocean-colour requirements from a fisheries and aquaculture perspective; and that by Sathyendranath et al. (2010) highlighted the need to integrate satellite with *in situ* observations to address the requirements for monitoring marine ecosystems. Le Quéré et al. (2010) placed the needs for ocean colour in the broader context of observational requirements for validating dynamic green-ocean models; and Claustre et al. (2010) articulated how ocean colour fitted into the need for an integrated sustained observation system for marine ecosystems and biogeochemistry. Finally, Drinkwater et al. (2010) in their discussion of the role of space observations in operational oceanography, drew attention to the importance of ocean colour for marine ecology and applications thereof.

The decade since OceanObs’09 has seen many reports that have refined and elaborated the user requirements for ocean-colour data and introduced additional requirements. They emerge from a variety of needs, including to understand the structure and function of the marine ecosystem and how it might be affected by climate variability and change, or by various types of pollution and their societal implications.

The areas of studies reported to benefit from sustained ocean-colour observations include:

- The global carbon cycle and its fluctuations at many time scales;
- Ocean acidification;
- Marine biodiversity and function;
- Validation and improvement of Earth System and ocean biogeochemical models;
- Data assimilation to improve model performance;
- Data for assessing impact and adaptation of marine ecosystem to climate change;
- Bio-feedback mechanisms, understanding Earth System;
- Flow of material through the marine food webs, implications for marine resources;
- Marine pollution; and
- The need to inform debates about bioengineering.

Notably, the IPCC Impacts and Adaptation Assessments (WGII) AR5 WGII Summary for Policymakers noted: ‘Open-ocean net primary production is projected to redistribute and, by 2100, fall globally under all RCP scenarios. Climate change adds to the threats of over-fishing and other non-climatic stressors, thus complicating marine management regimes (high confidence).’

The Global Climate Observing System (GCOS) defines a climate data record (CDR) as ‘a time series of measurements of sufficient length, consistency and continuity to determine climate variability and change’ and essential climate variables (ECVs) as measurements that contribute to characterisation of the Earth’s climate, for which a CDR may be created. Ocean colour is an ECV with two specific products: spectrally resolved remote-sensing reflectances or (normalised) water-leaving radiances and chlorophyll concentration. GCOS specifies requirements in terms of spatial resolution and uncertainties^3^ (Table 1).

The ESA Climate Change Initiative (CCI – see section ‘Time Series for Climate’) has been working toward producing ECVs that meet the GCOS requirements, and as part of the process has undertaken extensive user consultations. For ocean colour CCI the User Requirement Documents gave detailed requirements from a climate perspective^4^ revealing that in addition to the GCOS ocean colour ECV products, the user community has additional requirements for products that include **carbon products** and **phytoplankton types**. Improvements to **coastal algorithms** are also a requirement. The theme of carbon products from space was picked up by the CEOS ‘Carbon from Space’ Report (CEOS, 2014), which in turn was a response to the GEO Carbon Strategy Report. The CEOS report clearly recognises the central role played by ocean colour in generating carbon products (both **pools and fluxes of carbon**) in the aquatic environment (including marine and freshwater). It recognises the importance of integration at various levels: that of satellite observations with *in situ* observations and modelling; that across domains (land, water and air) given the importance of interfaces and **cross-domain fluxes**; the need to account for the role of coasts as the interface between land and ocean; and threeway coupling and feedbacks across domains. Another report worth mentioning in this context is the white paper on ‘Oceans and society: Blue Planet,’ which is a GEO initiative^5^that amalgamates the marine tasks within GEO. Many of the more recent reports have placed increasing emphasis on **data harmonisation, uncertainty characterisation, traceability and transparency**.

Considerations for identifying key variables have differed from report to report, and included factors such as the important environmental issues to be addressed, key questions to be answered, feasibility, cost, technology available for detection, platforms available for deployment, impact, and spatial and temporal scales of interest. However, the requirements for ocean-colour observations for **operational oceanography and for societal applications** have remained a common factor since POGO (Partnership for Observation of the Global Oceans) and CoML (Census of Marine Life) commissioned a report on ‘Biological observations of the global ocean: requirements and how to meet them’ in 2001. However, the products required from ocean-colour observations have grown over the years.

In addition to the products required from ocean colour, the uncertainty requirements merit consideration. For climate purposes, GCOS specifies (Table 1) 5% for water-leaving radiance and 30% for chl-a, taken to mean relative error related to bias (see section ‘Time Series for Climate’ and Figure 3). The OC CCI user consultation also sought input on uncertainties and responses were received from EO scientists engaged in global and regional analyses, trend analysis, primary production studies, fisheries, phenology and other applications, together with modellers working on global and regional models, validation/skill assessment, model development and data assimilation. A clear message from respondents (100% of modellers and 95% of EO scientists) is that product uncertainty estimates should be made available along with the product values and that uncertainties should be referenced to *in situ* data. Responses showed threshold (minimum) accuracy and precision requirements for chl-a of 10–25% (modellers) and 25–50% (EO scientists) with a goal of < 10%. This goal was considered to be equivalent to the uncertainty in *in situ* observation (CCI URD, 2014). However, within the EO and end-user communities the commonly *quoted* uncertainty requirement for chl-a is 35%. This is probably because the community is aware of the current capabilities of satellite EO observations where uncertainties of products are dependent on the uncertainties of retrieval algorithms, which in turn are based on linking *in situ* radiances and chl-a. each of which has its intrinsic uncertainties: this is discussed further in section ‘In situ Observations and Algorithms.’

Ocean colour is also a cross cutting Essential Ocean Variable (EOV) as defined by the Global Ocean Observing System (GOOS^6^), and EOVs are identified by GOOS Expert Panels on the basis of relevance, feasibility and cost effectiveness. Satellite ocean-colour measurements contribute to observations of a number of other EOVs including, *inter alia*: phytoplankton biomass and diversity, inorganic carbon, and dissolved organic carbon. Furthermore, analysis of ocean colour data can contribute understanding of other EOVs, such as frontal locations in relation to fish distributions. Hence, ocean colour is a vital component in the GOOS ‘Framework for Ocean Observing’ concept and, hence, to the aims of OceanObs19.

Ocean colour is relevant to the GEO Biodiversity Observation Network (GEO-BON) that has defined Essential Biodiversity Variables (e.g., Muller-Karger et al., 2018) as ‘the derived measurements required to study, report, and manage biodiversity change, focusing on status and trend in elements of biodiversity^7^.’ There are six EBV classes and 21 EBV ‘candidates’: the latter are linked to targets for the United Nations (UN) Convention on Biological Diversity (CBD^8^) as well as the UN Sustainable Development Goals (SDGs – see below). Ocean colour is of relevance to EBV class ‘Species traits’ candidate ‘phenology’ to observe global seasonal and interannual changes including bloom timing and duration, ‘Ecosystem function’ candidate ‘Net primary productivity’ and ‘Ecosystem structure’ candidate ‘Ecosystem composition by functional type.’

Finally, the UN SDGs provide a major global challenge^9^over the next decade. The SDGs have the aim of, *inter alia*, action to end poverty and economic inequality, address climate change, and sustainable consumption. There are 17 goals and the most relevant in the context of this review are Goal 14: ‘Life Below Water,’ while inland and coastal water colour and water quality is particularly relevant to Goal 6: ‘Clean water and sanitation.’ The SDGs are sometimes considered only with regard to developing countries, but whilst issues in many developing countries are acute, marine pollution, sustainable management of marine ecosystem, ocean acidification and increasing scientific knowledge are relevant to communities worldwide. Some training activities focusing on SDGs are presented in section ‘Training and Capacity Building.’

## OCEAN COLOUR EO SENSOR AND *IN SITU* OBSERVATIONS

This section provides an overview of current and future elements of ocean colour sensors together with relevant *in situ* data gathering to support ocean colour.

### EO Sensors With Ocean-Colour Capability

#### Polar-Orbiting Sensors

The first 15 years of the contiguous ocean colour record was characterised by one-off sensors or systems, like SeaWiFS, MERIS and MODIS (albeit with instruments on two spacecraft). By contrast, the next decade will see the availability of consistent observations from a series of missions supporting long-time series CDRs and operational monitoring applications, with processing by operational agencies such as NOAA in the United States and EUMETSAT in Europe. Complementing these systems are a number of current or planned missions that provide new capabilities (such as hyperspectral observations), geostationary platforms and systems that add to the CEOS ocean colour virtual constellation. These are all summarised in Table 2 and described below.

- In the United States:
  - The NASA **Aqua MODIS** instrument, launched in 2002, has provided a backbone for ocean colour observations over the past 16 years. With 9 bands in the visible/NIR and wide swath it provides coverage every day. Aqua-MODIS was planned to operate in constellation with Terra-MODIS launched in 1999 but sensor calibration of the latter limits its use in ocean colour applications (Franz et al., 2008; Kwiatkowska et al., 2008) and the long-term trend of Terra-MODIS has been linked to that of SeaWiFS and Aqua-MODIS and so is not recommended for use in CDRs.
  - The Joint Polar Satellite System (JPSS) programme comprises a series of launches of the **Visible Infrared Imaging Radiometer Suite (VIIRS)** instrument: the first on Suomi-NPP in October 2011 and the second on NOAA-20 in November 2017. Subsequent launches are planned on JPSS-2, 3 and 4 in 2021, 2026 and 2031, respectively^10^, all with the same instrument as SNPP and NOAA-20. VIIRS does not have some of the wavebands recommended by the IOCCG for ocean-colour sensors (International Ocean Colour Coordinating Group [IOCCG], 2012) but data have been shown to be of sufficient quality for addition to CDRs such the European Ocean Colour Climate Change Initiative products (see section ‘Time Series for Climate’). In addition, VIIRS has three SWIR bands recommended by International Ocean Colour Coordinating Group [IOCCG] (2012) for deriving ocean colour products over turbid coastal and inland waters.
  - The **NASA Plankton, Aerosol, Cloud, ocean Ecosystem (PACE)** mission, currently scheduled for launch in late 2022, is planned to carry the Ocean Color Instrument (OCI) that will provide hyperspectral radiometric measurements between 340- and 890-nm at 5-nm spectral resolution and at seven discrete bands in the short-wave infrared (940, 1038, 1250, 1378, 1615, 2130, and 2160-nm), with 1-day global coverage (2663-km swath) at 1-km resolution (nadir view). OCI is designed to support heritage and advanced ocean-colour applications, as well as atmosphere and cloud science. PACE is also expected to carry two polarimetres that measure polarised light at multiple view angles: the Hyperangular Rainbow Polarimetre 2 (HARP2), and the Spectro-polarimetre for Planetary Exploration (SPEXone). HARP2 is a wide-swath polarimetre (1556-km) that provide measurements at four discrete wavelengths in the visible to near infrared, and at 20–60 along-track viewing angles. SPEXone is a narrow-swath (106-km at nadir) hyperspectral polarimetre measuring from 385 to 770 nm with 2–4 nm spectral sampling and 5 along-track viewing angles. In addition to supporting cloud science and detailed aerosol property retrievals, the combined capabilities of the PACE polarimetres^11^ is expected to contribute to improvements in atmospheric correction for OCI ocean-colour retrievals.
- In Europe, the Copernicus programme plans four Sentinel 3 spacecraft carrying the **Ocean and Land Colour Imager** (OLCI: Donlon et al., 2012) with Sentinel 3A and 3B launched, respectively in 2016 and 2018. The motivation for Copernicus is to ensure continuity and consistency of observations enabling construction of services utilising these EO data. OLCI has 21 spectral bands from 400 to 1020 nm in the SWIR. Copernicus has an open data policy and supports upstream data processing; for OLCI processing of data from L1 to L2 will be undertaken by EUMETSAT and data are available via ftp and satellite digital broadcast (Eumetcast) making the data readily available even in developing countries with poor internet connections. Level 3 and regional (European) products are provided through the Copernicus Marine Environment Monitoring Service while the CDRs are available from the Copernicus Climate Change Service.
- In India, ocean-colour missions started with the launch of Oceansat-1 (on IRS-P4) with the **Ocean Colour Monitor (OCM)** in 1999 followed by Oceansat-2 in 2009 carrying a modified OCM-2 sensor. OCM is in operation and providing data with a spatial resolution of 360 m and global data at 1 km spatial resolution and temporal resolution of every 2–3 days. The OCM sensor has been used extensively for studying ocean phytoplankton, suspended particulate matter (SPM), and aerosol optical depth over ocean and data are operationally used for potential fishing zone selection (see section ‘Operational Ocean-Colour Applications’). OCM-3 is due for launch on Oceansat-3 in 2020.
- In Japan, the Japan Aerospace eXploration Agency (JAXA) launched the polar-orbiting **Global Change Observation Mission – Climate (GCOM -C)** satellite in December 2017 carrying the **Second-generation GLobal Imager (SGLI)**, a successor of the Global Imager (GLI) on-board the ADEOS II satellite. SGLI observes in 11 nadir channels in the visible – near-IR, with spatial resolution of 250 m, including the first observations at 380 nm, together with two polarisation forward/back along-track slant views channels at 673.5 and 865.5 nm with 1-km resolution. The initial plan for GCOM-C included the intermittent launch of 3 identical or similar satellite sensors over 15 years for a continuous monitoring of ocean colour.
- InChina, the civil space infrastructure development plan (2015–2025) foresees the launch of more than 15 ocean satellites, which includes ocean-colour satellites (**HY-1 series**), ocean-dynamics satellites (HY-2 series), and high spatial or temporal resolution monitoring satellites (HY-3 series). Three ocean-colour satellites (HY-1A, HY-1B, and HY-1C) have been launched, the last in 2018, and HY-1D will be launched in 2019. HY-1C and HY-1D, both with global ocean-colour observing capability, will be combined as a constellation observing in the morning (10:30 AM) and afternoon (1:30 PM), respectively. In addition to the sensors on HY-1B (Chinese Ocean Colour and Temperature Scanner and Coastal Zone Imager), HY-1C also has a new ultraviolet imaging sensor on-board, with 2 bands at 345–365 nm and 375–395 nm (550 m pixel resolution), which will be of benefit for atmospheric correction over turbid waters and for monitoring of CDOM. Ocean-colour satellites HY-1E and HY-1F, planned to be launched in 2021, will carry ocean-colour sensors similar to MERIS and OLCI, with high signal-to-noise ratios. The Moderate-resolution Wide-wavelengths Imager (MWI) on-board the Chinese Tiangong-2 Space Lab was launched in 2016, as an experimental precursor to HY-1E and HY-1F. With 100 m spatial resolution and 18 bands in the visible light and infrared wavelengths, MWI has proven the high quality of its ocean-colour observations, especially over coastal and inland waters (He et al., 2017).
- In Argentina, the Satélites Argentino-Brasileño para Información Ambiental del Mar (Sabia-MAR) mission is planned for launch in 2022 carrying optical sensors in the VIS/NIR/SWIR with 200 m resolution (regional) and 800 m (global). It was originally part of a two-satellite mission but the Brazilian spacecraft is unlikely to proceed, according to current status.

#### Geostationary Sensors

- Geostationary (GEO) ocean colour sensors offer the potential to observe variations in water optical properties over a day or a tidal cycle as well as mitigating the effects of clouds through repeated observations (see review by Ruddick et al., 2014). However, to date only the Korea Institute of Ocean Science and Technology (KIOST) **Geostationary Ocean Color Imager (GOCI)** instrument launched in June 2010 with 8 VIS/NIR bands at 500 m resolution has provided GEO ocean colour capabilities (Choi et al., 2012, 2014a). Neither of the proposed European GeOCAPI or US GEO-CAPE missions have progressed beyond initial planning. GOCI-II is scheduled for launch in 2019 on NEO-KOMPSAT-2B, possesses 13 VNIR bands at 300 m resolution and has Full Disk mode (providing one image per day) in addition to the Local Area mode of GOCI. China plans to launch a GEO ocean colour satellite, HY-3C, in 2022 with the prototype GEO ocean-colour sensor with 10 bands from 412 to 1640 nm (8 VIS/NIR; 2 SWIR), signal-to-noise ratio higher than 600, 250 m spatial resolution for VIS/NIR and 1 km for SWIR, and hourly observation over 2500 km × 2500 km (25 slots, each slot 512 km × 512 km). He et al. (2018) have developed a vector radiation transmission model for a coupled ocean-atmosphere system that takes into account Earth’s curvature (PCOART-SA), which can be applied to the high solar zenith angle satellite data from geostationary satellites (e.g., dawn and dusk observations).
- Geostationary meteorological sensors: the European Meteosat Second Generation **SEVIRI** was designed for meteorological applications, but with a single visible band has demonstrated capability to observe SPM or turbidity dynamics (Alvera-Azcarate et al., 2015; Kwiatkowska et al., 2016; Ody et al., 2016) and has been used in synergy with polar-orbiting ocean colour sensor observations (Vanhellemont et al., 2014). The Japanese Himawari 8 has 6 vis/NIR spectral bands including blue, green and red channels and can retrieve chl-a if data are averaged over 1 h (Murakami, 2016). Likewise, the forthcoming Meteosat Third Generation meteorological-focused Flexible Combined Imager has three visible bands and simulations have shown chl-a retrieval capability if data are averaged in space and time (Lavigne and Ruddick, 2018).

#### Novel Observation Platforms

- The European TROPOspheric Monitoring Instrument (TROPOMI) is carried on board the Copernicus Sentinel-5 Precursor satellite, the first of the atmospheric composition Sentinels, launched in October 2017. TROPOMI, similar to SCIAMACHY on the ESA ENVISAT platform (2002–2012), has ocean-colour capability, notably of phytoplankton functional types, through processing of the hyperspectral top-of-atmosphere radiance (e.g., Losa et al., 2017). Similarly, hyperspectral data from the Global Ozone Monitoring Experiment-2 (GOME-2) instrument carried on EUMETSAT’s MetOp series has been used to retrieve solar induced fluorescence (SIF) from chl-a (e.g., Joiner et al., 2016).
- NASA’s Earth Polychromatic Imaging Camera (EPIC) onboard the Deep Space Climate Observatory (DSCOVR) satellite, launched in 2015 and located 1.5 10^6^ km above the Earth’s surface orbiting the Lagrange point 1, provides repeated colour images of the Earth’s surface (its position is fixed with respect to the Earth and Sun). It possesses 10 narrow bands including three of ocean-colour relevance, but the pixel size at nadir is 8-km and the sensor signal-to-noise is only 200:1, with the consequence that it does not meet requirements for most ocean-colour applications. Nevertheless, Gao et al. (2019) produced atmosphere-corrected reflectances over various inland and ocean sites and described requirements for an ocean-colour capable sensor located at Lagrange 1.
- EUMETSAT’s MetOp second generation A missions will carry the Multi-Viewing Multi-Channel Multi-Polarisation Imaging (3MI) instrument with launches planned in 2022, 2029, and 2036. This mission complements the NOAA JPSS described above. 3MI has a secondary objective of ocean-colour observations, though it does not possess an on-board calibration facility and has relatively low SNR in its 12 VIS/NIR/SWIR spectral bands.

#### Higher Resolution Sensors

- Higher-resolution sensors are summarised in Table 3. Some, such as the United States Geological Survey Landsat 8 OLI and Copernicus Sentinel 2 MSI at 10–60 m resolutions were designed for terrestrial applications but have inland-water colour (e.g., Bresciani et al., 2018) or ocean-colour capabilities such as for monitoring suspended particulates (e.g., Vanhellemont and Ruddick, 2014; Vanhellemont and Ruddick, 2015) or coastal floating vegetation (Dogliotti et al., 2018). Very high-resolution sensors, some commercial, have been exploited for coastal monitoring of underwater vegetation, coral reefs, etc.
- Nano-satellites (or cube-sats) offer potential to create relatively low-cost ocean-colour constellations, and the proof-of-concept HawkEye instrument launched in December 2018 is the first of two sensors^12^ offering multispectral ocean colour observations in 8 bands with a sensitivity similar to SeaWiFS. The 120 m resolution and baseline of 15 scenes per day (each 200 km × 600 km) leads to a coastal or inland water observing paradigm, but global observing capability could be provided with a swarm of instruments.
- Finally, High Altitude Pseudo-Satellites (or High Altitude Platforms: HAP) are aircraft flying ~20 km high in the stratosphere, and may be balloons, airships, gliders or powered ‘planes, either manned or unmanned (d’oliveira et al., 2016). At such heights HAPs provide repeated observations similar to GEO spacecraft, but over a smaller range and at a much lower cost. A particular advantage is that missions would be typically of shorter duration and so HAP-borne instruments could be re-calibrated regularly. A number of concepts are under investigation (d’oliveira et al., 2016): disaster management or emergency response, or marine traffic situational awareness, form typical proposed environmental monitoring applications. In the ocean-colour field, monitoring for eutrophication or harmful algal blooms could be potential applications in areas of dense aquaculture or in response to a specific pollution event.

These sensors and applications, however, deserve a dedicated review paper, and so for brevity are not discussed further herein.

#### Adequacy of Ocean-Colour Missions

It is clear from the sections above that there are many ocean-colour missions in existence or in planning phase. This section reviews the adequacy of the missions for different scientific and operational needs. However, adequacy depends significantly on the nature of the application.

**Polar-orbiting** missions provide the mainstay for observational needs for scientists, for operational applications and monitoring purposes. The virtual constellation of two operational Sentinel 3 OLCI and two S-NPP/JPSS VIIRS together provide two observations per day at slightly different overpass times, albeit reduced by cloud cover. These are complemented by older sensors such as Aqua-MODIS or newer missions like GCOM-C.

For **climate applications** the key requirements are longevity and consistency of the ocean-colour record, with biases between the sensors needed to provide a record of sufficient duration, adequately characterised – this is discussed further in section ‘Time Series for Climate.’

**Operational applications**, such as monitoring for harmful algal blooms, are also served by the virtual constellations, with the multiple sensors providing gap-filling capabilities. It is important to note that the development of operational applications, notably by commercial companies, relies on the continuity provided in operational missions: that is to say, it would be difficult to justify commercial investment based on a single mission that may not be replaced. It is also worth noting that some applications do not need highly accurate ocean-colour estimates but instead use boundaries or gradients in water masses [e.g., see section ‘Use of Ocean Colour Data for Location of Potential Fishing Zones (PFZ)’].

Regarding **spatial resolution**, OLCI provides full ocean-colour capability to 300 m but higher resolution (of order 10–100 m) to detect in bays or estuaries, where natural or farmed aquaculture is often located, or the observation of colour in inland waters, are not adequately supported, since missions providing such data are focused on land applications (e.g., Sentinel 2 or Landsat 8). However, as noted above, sensors on these missions have limited capabilities for retrieval of SPM concentrations, and in some cases of chl-a concentrations. Mission requirements to meet these needs were presented in International Ocean Colour Coordinating Group [IOCCG] (2018). Improvements to the capability of Sentinel 2 or Landsat 8 sensors could partly meet these requirements, along with HAP or nanosatellites, assuming that issues of calibration are addressed.

Applications requiring **high temporal frequency**, such as to observe HAB species that migrate to surface waters during the day, require GEO ocean-colour sensors that currently are only available over east Asia (with GOCI – see section ‘Geostationary Sensor Monitoring of a Harmful Algal Bloom’). Conversely, commercial EO companies are addressing the need for frequent observations using constellations of satellites (Muller-Karger et al., 2018); however, these authors describe the stringent requirements for such a constellation designed for ocean colour observations, in terms of radiometric quality (high signalto-noise) and spectral resolution as well as spatial and temporal resolution.

Applications requiring **hyperspectral observations** are also not served by operational missions, though the launch of NASA PACE will support this area.

### *In situ* Observations and Algorithms

To infer in-water properties such as chl-a from satellite observations of ocean colour requires retrieval of the water-leaving radiance that, as noted above, may be less than 10% of the top of atmosphere detected signal and, hence, requires high precision in the satellite sensor calibration. Then, the retrieved water-leaving radiances are linked to in-water properties using models or algorithms (harnessing new capabilities when available, e.g., hyperspectral measurements). Radiometric calibration of spaceborne sensors was addressed by IOCCG report 14 (International Ocean Colour Coordinating Group [IOCCG], 2013) and various recommendations were made, including pre-flight characterisation of sensors; calibration of reflectance instead of radiance; vicarious calibration to meet accuracy requirements (see below); the location of calibration sites, i.e., spatially homogenous oceanic regions sufficiently far from land; consistency in approaches between space agencies; and easy availability of calibration data to the ocean colour community.

Regarding *in situ* data for algorithm development or calibration/validation of satellite-retrieved geophysical products, the *in situ* measurements must meet strict criteria, including being taken using instruments with documented SI traceability based on metrology standards and an associated uncertainty budget, collected following community-agreed protocols and procedures, and freely available to other researchers for independent verification. Such data have been termed Fiducial Reference Measurements (FRM). Recommendations on the need for *in situ* measurements for satellite calibration and validation of long-term CDRs were documented in a white paper by the International Network for Sensor Inter-comparison and Uncertainty assessment for Ocean Color Radiometry (INSITU-OCR) initiative^13^ and are updated as part of the on-going activities of the Ocean Colour Radiometry – Implementation Team (OCR-IT). Protocols for *in situ* observations were produced during the SeaWiFS era and recent efforts and discussions at meetings such as the IOCS, have been made to update these protocols, taking into account developments in sensors and sampling platforms; draught and final texts are available at http://ioccg.org/what-we-do/ioccg-publications/ocean-optics-protocols-satellite-ocean-colour-sensor-validation/.

#### Algorithms

Algorithms link the satellite observations of water-leaving radiance (retrieved after atmospheric correction) and the in-water quantities of interest such as chl-a. Many algorithms have been proposed for chl-a, for example, and this field has been the subject of many review papers and is only covered briefly herein: readers can consult International Ocean Colour Coordinating Group [IOCCG] (2018) and forthcoming reports (such as the GEO AquaWatch review) for further details.

For ocean and shelf waters the most commonly used algorithms are band ratios of blue green wavelengths (e.g., O’Reilly et al., 1998) constructed by regression of *in situ* observations. A common feature of such algorithms, even in socalled ‘case 1’ waters where the optical properties are driven by phytoplankton and co-varying optically active substances, is the considerable variability around the regression. This is probably due to the diversity of water types encountered and a result of the uncertainties in the *in situ* measurements (e.g., radiances and chl-a) used in construction of algorithms. Furthermore, an *in situ* chl-a sample may be from 1L of water and is compared with *in situ* upwelling radiance observations representing a signal from a much larger mass of water.

A summary of studies comparing chl-a algorithms applied to single sensor or multi-sensor merged satellite records with global *in situ* datasets showed average root mean square error of 0.337 log_10_chl-a (Brewin et al., 2016). A further issue is that global analyses are usually based on measurements from independent investigators meaning that methodological differences between laboratories can introduce additional uncertainties. Sørensen et al. (2007) compared measurements of chl-a by high performance liquid chromatography (HPLC – considered the reference technique for chl-a) by 11 teams involved in the validation of the ESA MERIS sensor. Measurements showed wide variations amongst teams: in the first intercomparison using algal cultures the coefficient of variation (CV) spanned 10–25%; in a second intercomparison based on natural waters, CV was 10–16% in ‘case 2’ waters and 7–40% for case 1 waters. These results were after removal of ‘outliers’ which, in the field, may not be recognised as such. They attributed much of the difference to extraction methods since an intercomparison of chl-a extracts showed CV of 8–15%. Another intercomparison (Claustre et al., 2004) of four HPLC laboratories found a lower uncertainty (expressed as absolute percentage difference) of 7% for total chl-a across a wide trophic range. Furthermore, by applying further quality assurance procedures the uncertainty was reduced to ~5.5% for total chl-a. Both these studies reinforce the need to follow internationally agreed protocols as described above.

Another issue in the validation of satellite algorithms (and manifested in the results of a comparison of satellite retrievals with *in situ* observations) is the differences in scales of observations between satellite and *in situ* data. Applying an algorithm to average upwelling radiance observed over a pixel ~1 km^2^ with a nominal penetration depth of ~10 m, and comparing the result with an *in situ* observation based on 1 L of water represents a scale difference of 1:10^−10^. This raises concerns on how representative an *in situ* sample of a 1 km^2^ body of water is. This issue was investigated by Brewin et al. (2016) who used measurements from two Atlantic Meridional Transect (AMT) cruises between 50° N and 50° S of along-track particulate absorption calibrated against HPLC–chl-a to obtain multiple *in situ* measurements within a satellite ‘pixel.’ With this approach they found that the uncertainty in satellite retrievals reduced to 0.157 log_10_chl-a on average, which is less than half that reported from previous global studies. They suggested that the better performance may be due to the timing of the AMT cruises (in October-November 2009 and 2012) or the restriction to just the Atlantic (limiting the variety of waters encountered), but it could also reflect HPLC analysis at one laboratory (avoiding inter-investigator differences) using an accurate automated system that minimises human error, and also taking multiple measurements within an individual satellite pixel to better characterise the sub-pixel variability.

Rather than applying algorithms globally, another approach is to first classify individual pixels optically on the basis of their reflectance spectra (Moore et al., 2009) and then use the best-performing algorithm selected for each water class (Moore et al., 2014; Jackson et al., 2017). This approach is described in more detail in section ‘Time Series for Climate’ along with a view on the ability of algorithms to meet requirements.

#### *In situ* Observations to Calibrate Ocean-Colour Sensors and Evaluate Products

##### Radiometric calibration

As noted by International Ocean Colour Coordinating Group [IOCCG] (2013) ‘uncertainty requirements for scientific applications, e.g., 5% absolute in the blue and 1% relative (i.e., band to band), or 30% on chl-a concentration, radiometric calibration should be accurate to a fraction of 1%.’ However, despite careful pre-flight and post-launch calibration efforts such as periodically viewing on-board calibration sources or external targets such as the sun and moon (International Ocean Colour Coordinating Group [IOCCG], 2013; Zibordi et al., 2015) it has not been possible to retrieve radiances of sufficient accuracy. Hence, **System Vicarious Calibration (SVC)** of spaceborne sensors is undertaken whereby the retrieved satellite top of atmosphere radiance is compared with that expected from transmission of measured water-leaving radiance to the top of the atmosphere, using the same atmospheric model used for the in-water radiance retrieval. It is described as a *system* calibration since it incorporates the effects of the entire processing chain and is therefore specific to individual atmospheric correction procedures. The NOAA operated Marine Optical BuoY (MOBY: Clark et al., 1997) system located off Hawaii in very low chl-a water is used for SVC, along with the French Boussole system in the Mediterranean, though SVC can also utilise other systems (such as AERONET-OC). Maintenance of MOBY and other SVC systems is a key continuing requirement: in addition to continued support of MOBY by NOAA in the United States, EUMETSAT is conducting projects to develop SVC capability in Europe.

Ongoing high-quality *in situ* radiometric measurements are also needed to validate new sensors in a satellite constellation as well as to test continually the performance of instruments that have been in orbit for many years. This is important since the premature addition of a new sensor to a CDR before inter-sensor biases are accounted for, or failure or degradation of an older instrument, may introduce spurious trends into the record (e.g., Brewin et al., 2016). Likewise, for operational applications a new sensor with a bias could potentially lead to spurious alerts for events such as eutrophication. The AERONET-OC initiative provides quality-controlled multispectral data for a number of fixed locations worldwide (Zibordi et al., 2009), which is likely to be complemented by a hyperspectral observation network, for example, the recently started European Hypernet project^14^. Bio-optical instruments such as above-water radiometers, and flow-through absorption/attenuation instruments, are increasingly being installed on moving platforms such as ships of opportunity, research vessels, or even yachts of environmentally aware philanthropists. Quality control of along-track data is vital (see IOCCG page on protocols) but such systems have shown considerable potential to increase significantly the number of satellite validation matchups (Brewin et al., 2016; Dall’Olmo et al., 2017).

##### In situ matchup database

For satellite validation and SVC it is important to have access to the various sources of data in one place. The ESA Ocean Colour CCI project has constructed such a database with global distribution that includes data acquired from several sources: a version was published as a paper/dataset in Valente et al. (2016) and is also available at PANGAEA^15^. The latest version (v4) comprises data from 1997 to late 2017 on spectral remote-sensing reflectances, concentrations of chl-a, spectral inherent optical properties, spectral diffuse attenuation coefficients and total suspended matter.

##### The argo float network

The argo float network comprises 4000 floats worldwide measuring vertical ocean physical structure. The network of Biogeochemical-Argo (BGC-Argo^16^) floats is smaller, comprising hundreds of floats, carrying a variety sensors to measure oceanic biogeochemical properties such as chl-a fluorescence, oxygen pH or nitrate (e.g., Dall’Olmo and Mork, 2014; Dall’Olmo et al., 2016). In principle, BGC-Argo-measured chl-a could provide the depth dimension not sensed by ocean-colour satellites but this is complicated by chl-a fluorescence quenching near the surface, though Xing et al. (2011) have proposed correction of fluorescence using irradiance sensors on the float. There are also various physiological processes that contribute to complex relationships between chl-a fluorescence and concentration, such that interpretation of the fluorescence signal as concentration is not straightforward. BGC-Argo data on spectral diffuse attenuation of downward irradiance, fluorescent dissolved organic matter concentrations, and particulate optical backscattering have been compared with satellite retrievals (Organelli et al., 2017). Upwelling radiance sensors on BGC Argo floats (Leymarie et al., 2018; Wojtasiewicz et al., 2018) offer potential for validation of EO sensors, though long-term calibration and bio-fouling may impact upon the performance.

### Citizen Science

Citizen science or crowdsourced environmental observations is an area that has grown rapidly and is likely to grow further over the next decade as the worldwide usage of smart phones increases. Citizen science may provide useful contributions to ocean-colour observations, albeit primarily in coastal and inland waters as well as raising environmental awareness. Examples of ocean-colour relevant citizen science projects include:

- The Secchi Disk Project^17^ is the largest worldwide citizen science study collecting measurements of ocean and coastal transparency with the Secchi disk (Lavender et al., 2017). A phone app is used to geo-locate and record the Secchi disk measurements, which are subsequently transmitted to a data repository.
- Citizen science measurements using a 3D-printed mini Secchi disk (Brewin et al., 2019) are contributing to a research project monitoring water quality (visibility and colour) of waters of Lake Vembanad in India, for comparison with satellite products of the region, contributing to a United Kingdom–India jointly funded research project.
- The EyeOnWater^18^ mobile phone app was originally developed in a European citizen science project (Citclops) that enables the user to classify a water body using the Forel-Ule 21 water-type colour scale that is displayed on the smartphone screen. Data are then uploaded to a central repository.
- The BloomWatch app^19^ documents the location and frequency of potentially harmful cyanobacteria blooms. The user is prompted through a series of screens to provide site information and photos.

## GROUND SEGMENT AND DATA PROCESSING

### Ground Segments

The term ground segment is used loosely herein to define the systems that include reception of EO data *via* satellite downlink and lower-level processing for calibration and geolocation, followed by atmospheric correction and production of analysis-ready data and end-user focused services; it also includes timely and appropriate delivery mechanisms (International Ocean Colour Coordinating Group [IOCCG], 2018). The ground segment is an area that has, arguably, improved considerably over the past 10 years and, indeed, the success of an EO mission, once launched, is ultimately determined by the use of the data, information and services by stakeholders for applications. Below we describe example ground segments.

#### Copernicus

Copernicus is a flagship European programme that incorporates Earth Observations encompassing satellite constellations (the Sentinel series, notably Sentinel 3 – see section ‘Ocean Colour EO Sensors and in situ Observations’); ground-segments producing operational products and CDRs; and training and outreach activities. Copernicus has an open data policy whereby data can be used free of charge for any purpose, academic or commercial. For ocean-colour data from the OLCI sensor, the basic data processing from L0 to L1 and L2 is undertaken operationally by EUMETSAT. Users can also obtain the L1 and L2 data and process using tools such as the Sentinel Application Platform (SNAP^20^), provided through the ESA which offer additional capabilities, such as different atmospheric correction procedures. Copernicus supports a marine-focused processing service, the Copernicus Marine Environment Monitoring Service (CMEMS). The CMEMS ocean-colour team uses OLCI, VIIRS and Aqua-MODIS data to produce near-real time and reprocessed time series of global mapped products, and regional products with bespoke, regionally tuned algorithms, such as for the Mediterranean Sea (Volpe et al., 2007). CMEMS produces ocean monitoring indicators (OMIs) and publishes annual ocean state reports which include ocean-colour results (von Schuckmann et al., 2016, 2018 see below). Copernicus envisages that ‘downstream’ services addressing individual end-user or application sectors are produced by national agencies or private companies building on the CMEMS ‘upstream’ products. An ocean-colour CDR is produced by the Copernicus Climate Change Service (C3S) and brokered to CMEMS. Although Copernicus services are operational and do not explicitly support R&D, the products are maintained at the state-of-the-art and so incorporate the latest R&D: in the case of C3S the CDR benefits from R&D in the ESA CCI project (see section ‘Time Series for Climate’). Finally, training in ocean-colour data is undertaken through, *inter alia*, the EUMETSAT marine training programme that includes European and international elements, the latter primarily, but not exclusively, in developing countries.

#### NASA OBPG

The NASA GSFC Ocean Biology Processing Group (OBPG) produces ocean colour data from NASA sensors such as CZCS, SeaWiFS and MODIS, as well as third-party missions including NOAA’s VIIRS, ESA’s MERIS (both reduced and full resolution, and operated 2002–2012) and the Korean GOCI. All source data and derived data products are freely distributed through the Ocean Biology Distributed Active Archive Center (OB.DAAC) and directly accessible via the Ocean Color Web portal^21^. Data are provided at Level-1: raw or calibrated and geolocated observations at native resolution; Level-2: derived geophysical variables at native resolution; and Level-3: derived geophysical variables that have been aggregated/projected onto a well-defined spatial grid over a given time period, including daily, weekly, monthly, seasonal and annual composites at 4.6 or 9.2-km spatial resolution (depending on sensor). Individual images can be downloaded as PNG or NetCDF files, and orders can be placed for bulk downloads with subsetting options. For the global missions supported by the OBPG, a primary focus is on the use of consistent algorithms and calibration approaches to produce a continuous time-series of standardised ocean colour products for global change research and Earth system science applications.

#### NOAA

Data from both VIIRS sensors are processed by NOAA to produce global maps^22^ and merged VIIRS products are under development. Data are also produced in near-real time for different user regions-of-interest via CoastWatch^23^. In addition, near-real time ocean-colour data compared with *in situ* data from MOBY and several AERONET-OC sites are available through the NOAA website. All VIIRS global ocean-colour data are freely available through NOAA CoastWatch website.

### Data Delivery

**FIGURE 1 |:**
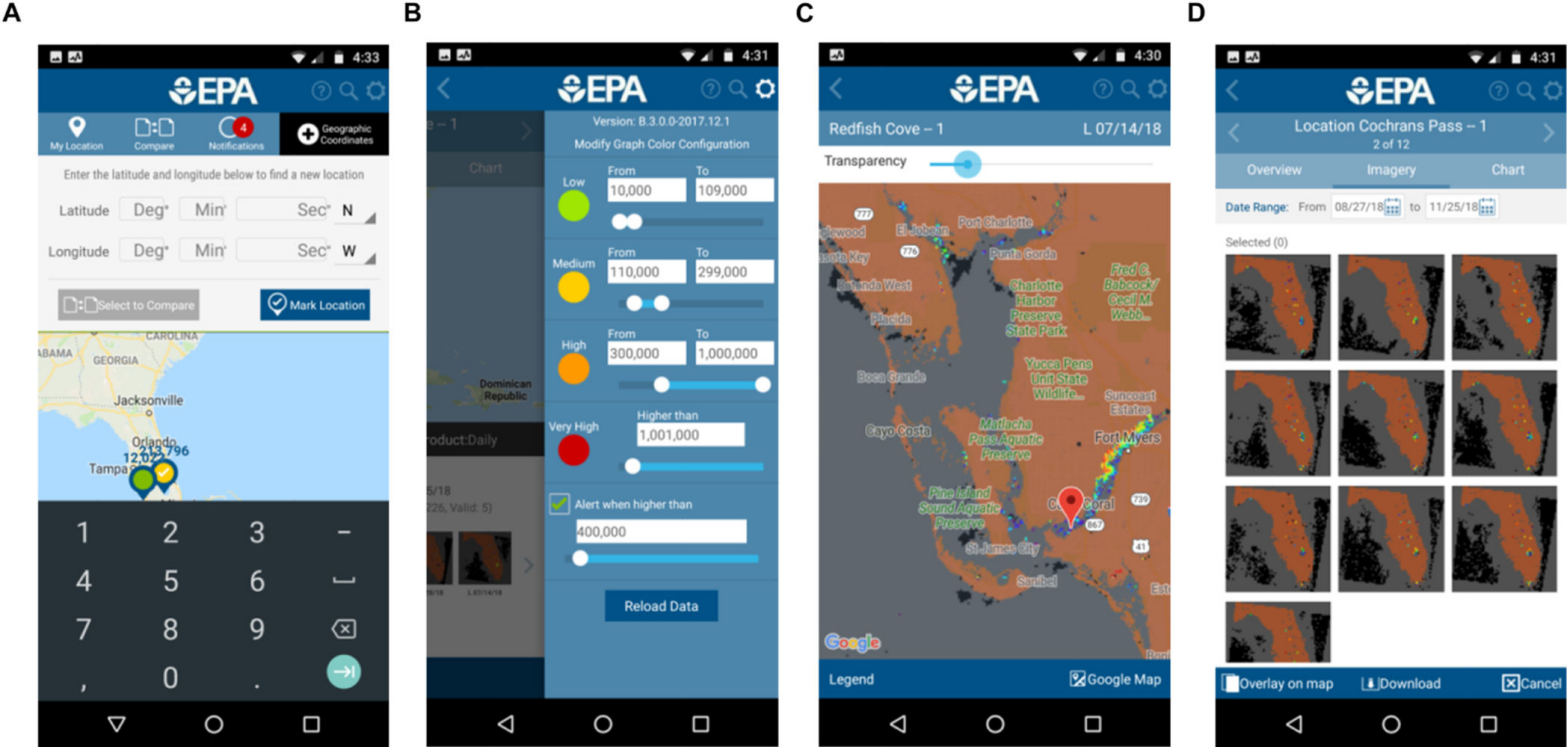
Screenshots of CyAN mobile app (Schaeffer et al., 2018): (**A**) The main page of the app allows for dropping locations of interest pins using latitude and longitude coordinates or by responsive touch on the screen. (**B**) The user is also able to set pin colour thresholds and an alert icon setting with the cogwheel based on their own criteria. (**C**) selecting a pin allows the user to visualise the complete satellite tile for the location of interest for spatial patterns, such as demonstrated with a coastal estuary on the west Florida shelf where Google maps is a transparent layer for quick geographic reference. (**D**) The user can view a temporal series of images related to their pin location and visualise temporal trends amongst different locations, where each line graph is a single pixel (not shown).

**FIGURE 2 |:**
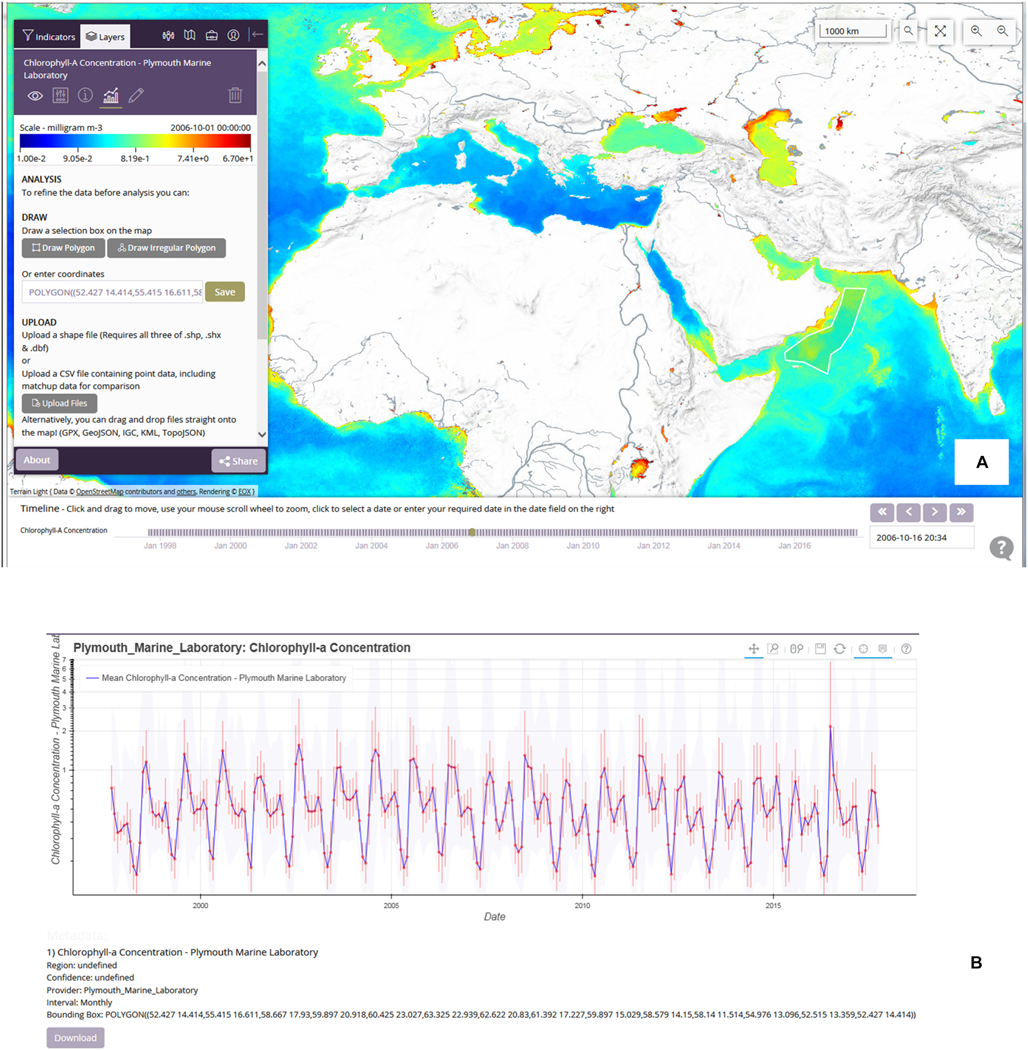
Screenshots for ESA ocean colour CCI OGC-compliant visualisation/analysis portal: **(A)** monthly version 3.1 global chl-a map (comprising merged bias-corrected SeaWiFS, Aqua-MODIS, MERIS and VIIRS) with uploaded shapefile for image data extraction. **(B)** Time series plot for region of interest; vertical bars show standard deviation and background grey shows range.

- Traditional methods such as ftp and web-downloads, e.g., via the NASA browser noted above or the ESA Ocean Colour Climate Change Initiative site^24^, will continue to be exploited by many users.
- GEONETCast is a distribution system based on Digital Video Broadcast technology with multicasting of environmental data via commercial satellites. It is particularly valuable for institutes or universities in developing countries with poor internet access due to their location, such as marine institutes located at the coast, or due to limited national infrastructure. For Africa, two European Commission funded projects installed low-cost GEONETCast receivers at sites across the continent. The systems had mixed success, not least due to the exposed locations at the coast. The Monitoring of Environment and Security in Africa programme (MESA^25^: completed in 2017) installed further reception capacity based around Regional Implementation Centers. Ocean colour data are still transmitted via the Eumetcast and data provision is being maintained in the ‘GMES and Africa’ project.
- Mobile applications. According to the World Bank in 2016 there were more mobile cellular subscriptions than people in the world^26^ while by 2019 it is forecast that 63% of the population will have mobile technology with internet connectivity^27^. Indeed, in Africa, mobile coverage has enabled ‘...Africans to skip the landline stage of development and jump right to the digital age’ (Pew Research Centre, April 2015). Mobiles offer great potential for dissemination of ocean-colour data for a variety of applications. It obviously opens up two-way conversation, encouraging citizen scientists (see section ‘Citizen Science’). Example mobile apps focusing on ocean and inland colour applications include:
  - The CyAN mobile app. Most water quality managers and local interest groups do not have time for extensive training in building a technical background for deriving and processing satellite derived water quality data and information. Therefore, a passive software system may benefit end-users that limit this technical burden and time commitment. Schaeffer et al. (2018) demonstrated an operational mobile application (see Figure 1) that allows passive reception of Copernicus Sentinel-3 OLCI data instead of active acquisition minimising the barrier to receiving actionable information (Schaeffer et al., 2013). The app functionality was successfully replicated across 25 individual state health advisories in 2017, proving it could provide a user-friendly platform reducing complexity traditionally associated with using satellite data (Schaeffer et al., 2018). Currently, a number of state environmental and health departments, non-government organisations, and some local interest groups are using this application across large lakes and coastal regions.
  - The ABALOBI mobile app^28^ is planned to be used by the GMES and Africa southern Africa centre to deliver Potential fishing zone products. PFZ information is already supplied to Indian fishers through a mobile from INCOIS (see section ‘Operational Ocean-Colour Applications’).
- Web-based analysis tools. For exploitation of ocean-colour data by non-specialist end-users it is important to provide easy-to-use tools, ideally web-based, that support simple analysis of datasets provided by the ground segments. This ensures that EO data have an impact beyond specialist academic communities. Such a system should be built ideally on recognised standards and allow sharing of data or visualisation of data from multiple providers. The Open Geospatial Consortium (OGC) specifies standards for accessing data via the web including Web Mapping Service (WMS) serving images (such as GIF), Web Coverage Service (WCS) which serves arrays of data and Web Feature Service (WFS) that specifies point or other vector data such as shiptracks or region of interest boundaries. In principle, web-based portals using these standards can request and visualise data from other portals. Many portals have built-in analysis capabilities, such as time-series plotting; this is also efficient since only delivery of the results is required, not the full dataset from which it was derived and which may comprise many gigabytes or terabytes of data. An example of a web-vis system is the ocean colour CCI portal^29^(see Figure 2A) which allows selection of regions of interest (e.g., via hand-drawn polygons or uploaded shapefiles) then produces time series, Hovmöller plots, or time animations of these data. Figure 2B shows an extracted time series of chl-a from a user region of interest. The portal also provides additional analyses such as scatterplots between two variables, and data extraction in time and space, from an uploaded location/date file, such as along a ship track. Hence, it can be used to for satellite matchup analysis. Another important ocean-colour tool is GIOVANNI as provided by NASA^30^.

### Cloud Platforms

The increase of data volumes resulting from new higher resolution or hyperspectral sensors and the growing length of the ocean-colour record is creating challenges for data processing, and even more so for accessing the data. Bandwidth can inhibit systematic near-real time processing at a global scale, or analysis of a complete archive. In recent years, there has been a paradigm shift from downloading and processing data locally, to moving the processing software at the data source, together with provision of data processing resources. This is an ongoing process and most users still download data and process in the traditional way. This may be attributed to the fact that specialised systems, i.e., hardware and software with cost implications, are required to support the new way of processing EO data close to the data source as well as conservative practice. The Copernicus **Data and Information Access Services (DIAS)** are planned to facilitate access to data and information from the Copernicus services. By providing data and information access alongside processing resources, tools and other relevant data, this initiative is expected to boost user uptake, stimulate innovation and create new business models based on EO data and information. More information can be found at^31^.

Accessing_Copernicus_data_made_easier. **ESA’s Thematic Exploitation Platforms (TEPs)** concept aims to provide a working environment where users can access algorithms and data remotely, providing them with computing resources and tools that they might not otherwise have, and avoiding the need to download and store large volumes of data. Currently, there is a coastal TEP focusing on coastal ocean-colour applications. Finally, various commercial suppliers like Amazon Web Services offer cloud computing with EO data series.

## TIME SERIES FOR CLIMATE

Ocean-colour CDRs of sufficient duration to quantify natural variability and to detect climate change impacts on the marine ecosystem are the key requirement emerging from user consultations. Henson et al. (2010) showed that a contiguous recorded in excess of 40 years is needed to distinguish a climate-related trend in chl-a and primary production from natural variability, though only > 20–30 years is needed for chl-a in some regions such as equatorial waters. Furthermore, the minimum length of time series required increases in the event of gaps in the record. The uninterrupted ocean-colour record started in 1997 with SeaWiFS and so in 2018 is only 21 years; no individual sensor covers all of this period and in any case most sensors have finite lifespans, and older sensors exhibit calibration degradation. So, to create a continuous time series requires merging (International Ocean Colour Coordinating Group [IOCCG], 2007) data from individual sensors without introducing biases, artifacts or discontinuities (Hammond et al., 2017). However, the sensors launched over this period each have different characteristics in terms of orbits (overpass in the morning, mid-day or afternoon), different swaths (and, hence, re-visit times), spatial resolution (4 km to 300 m), and a different number and location of spectral wavebands. So construction of a multi-sensor merged time-series is not a trivial task.

ESA’s Climate Change Initiative (CCI; Hollmann et al., 2013) has been supporting the research to develop and validate algorithms, initially for 13 Essential Climate Variables (ECVs) including ocean colour, to produce consistent, stable, error-characterised global satellite data products from multi-sensor data archives, that meet the GCOS requirements (Table 1). Since 2010 the Ocean Colour CCI team has been working with support and help from NOAA and NASA to perform research and development, and undertake regular re-processing to extend the time series implementing the R&D (Brewin et al., 2015; Muller et al., 2015a,b; Sathyendranath et al., 2017). The team includes representatives of the global climate research community, including marine ecosystem modellers and remote sensing scientists, who provide ongoing feedback. Community consultation and participation have been cornerstones of this initiative.

To produce the ocean-colour CDR, MERIS, Aqua-MODIS, SeaWiFS, and SNPP-VIIRS remote sensing reflectances (R_rs_) are merged following band-shifting (Melin and Sclep, 2015) to a reference sensor (SeaWiFS) and pixel-specific inter-sensor bias correction. Bias climatologies between a sensor and the reference (e.g., MERIS and SeaWiFS) are computed using 5 years (2003–2007) of overlapping data, taking into account seasonal and regional variations (Bias correction of VIIRS uses a two-step process of shifting Aqua-MODIS to SeaWiFS and then VIIRS to Aqua-MODIS). The project used a novel atmospheric correction approach (Steinmetz et al., 2011) to better deal with areas of sunglint and haze: this is important since some areas such as the Arabian Sea (Al-Naimi et al., 2017) or Red Sea (Racault et al., 2015) previously had long periods of no coverage in some precursor multi-sensor merged or single sensor data sets. Such gaps also confound elucidation of long-term trends. Sensor-specific R_rs_ are then merged using simple averaging of bias-corrected, band shifted data.

To compute per-pixel uncertainty metrics (bias and root-mean-square difference, RMSD) and also to select the in-water retrieval algorithm, an optical water-type classification approach is used to compute pixel water type (Moore et al., 2014; Jackson et al., 2017). Satellite-derived radiances are used to construct a set of optical water types (OWT); then uncertainty metrics are computed per OWT through matchups between satellite R_rs_ and in-water constituent values from the OC CCI database (Valente et al., 2016). For each pair of satellite R_rs_ and *in situ* water constituent the pixel water class memberships (from the R_rs_) are used to weight the differences in computation of RMSD and bias. This approach was followed since the OC-CCI user consultation indicated a clear preference for uncertainties based on validation of the products against *in situ* data. Finally, for each pixel, uncertainty is computed from the per-class uncertainties using the pixel class membership (Jackson et al., 2017). Chl-a and other in-water variables are computed using class membership and the optimal algorithm per water class. Chlorophyll algorithms were selected from amongst OC4/OCI (Hu et al., 2012) and OC5 (Gohin et al., 2002) through round robin assessment utilising a scoring system similar to that of Brewin et al. (2015).

**FIGURE 3 |:**
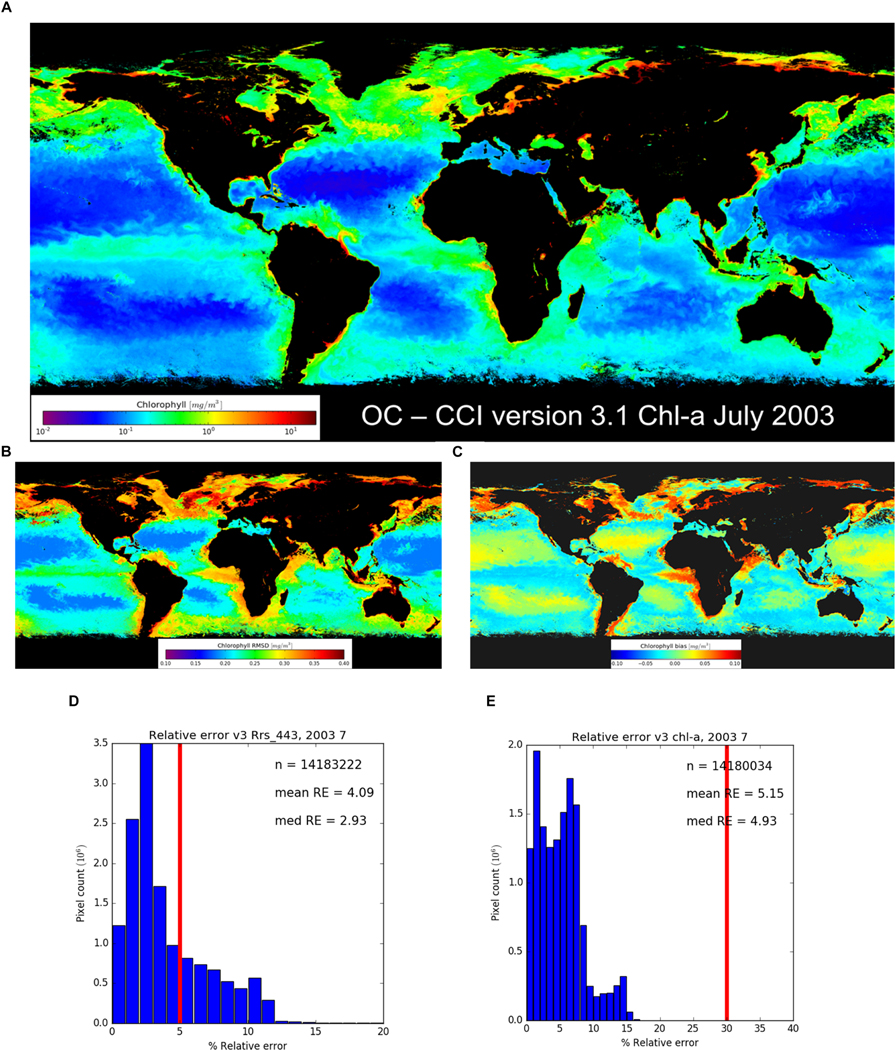
ESA Ocean Colour Climate Change Initiative version 3.1 monthly images for July 2003: **(A)** chl-a concentration, **(B)** RMSE, and **(C)** bias expressed as log10chl-a; comparisons of monthly relative error with GCOS uncertainty criteria for **(D)** R_rs_443 nm and **(E)** chl-a for July 2003.

Assessment of the OC CCI products has included suitability for use as a CDR (Melin et al., 2017); the impact of discontinuities on computing trends (Hammond et al., 2017); and comparison of OC CCI data with precursor datasets (Couto et al., 2016). Example results from OC-CCI version 3.1 (available at www.oceancolour.org) are shown in Figures 3A–C including a chl-a product, RMSD and bias expressed as log10chla. RMSD and bias vary with water class, with typically lower RMSD in water classes representing clearer open-ocean waters (Jackson et al., 2017).

OC CCI v3.1 data have been compared with the GCOS requirements for uncertainty (Table 1) by computing absolute per-pixel relative error (∈ = | 100 [1−(1/10^m^)] |) from bias (m) e.g., for chl-a, m is in units of log_10_(chl). In general, OC CCI products meet the GCOS requirements for uncertainty (see Figures 3D,E) though long-term stability is still under investigation.

**FIGURE 4 |:**
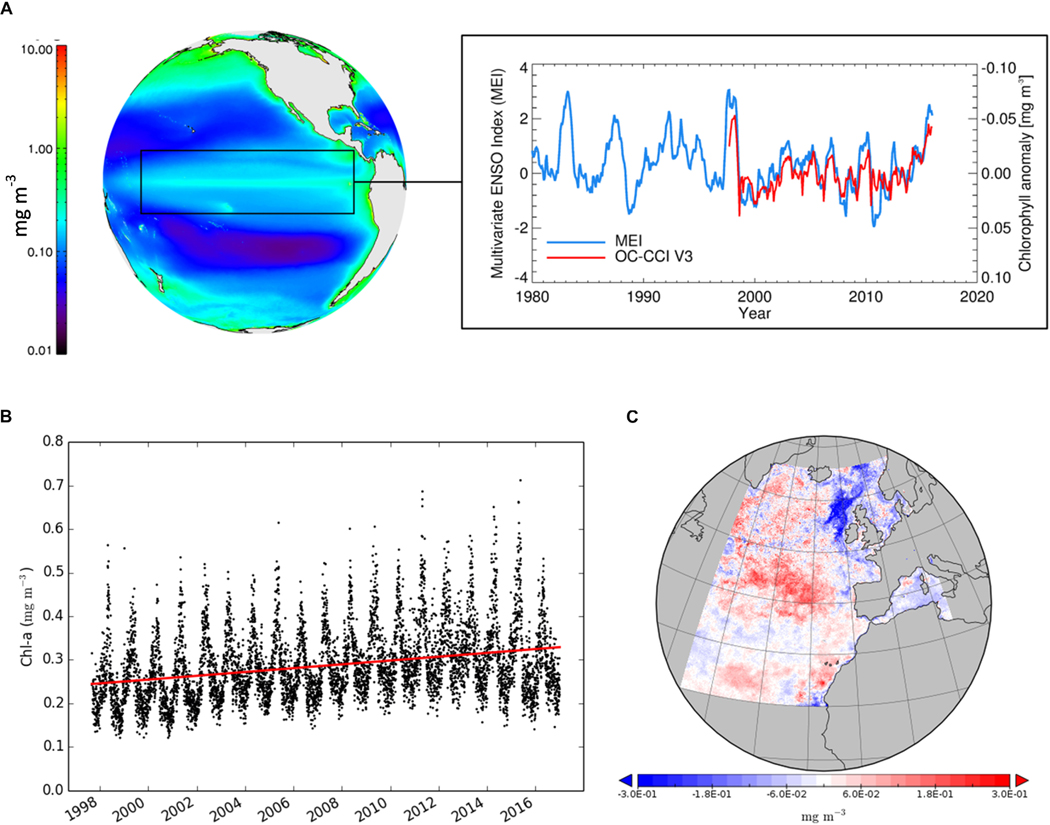
**(A)** Relationship between chl-a and the ENSO climate mode: note that the scale of chl-a anomalies is inverted. chl-a image is from an annual climatology. The monthly multivariate ENSO Index (MEI) was downloaded from the NOAA website (http://www.esrl.noaa.gov/); monthly plotted chlorophyll data were taken from the OC-CCI/CMEMS data set (modified from von Schuckmann et al., 2016); CMEMS north-east Atlantic region: **(B)** Time series (1997–2016) plot; **(C)** anomaly map for 2016 (with respect to 1997–2014 reference period) (re-worked from von Schuckmann et al., 2018).

Although the time series is too short to unambiguously detect climate change impacts (Henson et al., 2010), the CDR can be compared to climate indicators. Figure 4A from von Schuckmann et al. (2016) shows interannual variations in chl-a concentration in the Pacific Ocean and close correspondence with the ENSO index. This regional response to climate variability gives important clues on how phytoplankton might respond to long-term climate changes. The 2015–2016 ENSO event was the strongest observed since 1997. The OC CCI reflectance data are used in the CMEMS project with regionally tuned algorithms to provide optimal ocean-colour time series for European seas. Figure 4B from von Schuckmann et al. (2018) depicts the general trend in chl-a in the northeast Atlantic from 1997 to 2016 where the red line shows a simple linear fit to the data: no correction was made for outliers nor was any seasonal signal removed. The trend shows a steady increase until 2014–2016. An anomaly map (with respect to the 1997–2014 period) for 2016 (Figure 4C) for the northeast Atlantic shows positive anomalies except for European shelf waters, which show mostly negative anomalies.

Sathyendranath et al. (2017) described the characteristics of algorithms and methods needed for an ocean colour CDR. Amongst the 20 requirements are included: use of retrieval algorithms robust to changes in the environment; methods or algorithms should perform routinely and globally and should minimise gaps in data; inter-sensor bias needs to be corrected before multi-sensor merging; and algorithms should retrieve products with minimal uncertainties. Striving to meet these requirements needs additional research and development such as approaches to minimise gaps in the data, and improved coverage is expected through constellations of EO sensors. The next decade will see the time series extending to 30 years, which could allow investigation of climate impacts on the ocean ecosystems, for some regions, albeit with caveats on gaps and discontinuities (Henson et al., 2010). The similarity in sensor characteristics and orbits of the operational systems (Sentinel 3 OLCI and NOAA VIIRS) at least within each constellation should minimise discontinuities in the CDR caused by the introduction or removal of an individual sensor, whilst regularity in sensor launches should remove the issue of gaps, at least through lack of sensors, even if problems with extended cloud-cover may continue to pose problems. It is important that the Sentinel-3 OLCI-like series be continued well beyond the planned 20 years, to provide a baseline for long-term climate data records.

A further development will be the integration of the ocean-colour CDR with *in situ* data from Argo and BGC-Argo programmes, as well as other EO data in synergy, to characterise the 3D structure of the ocean. In particular, the BGC-Argo fleet is expected to expand, albeit not to the density of Argo, but with backscattering sensors (Dall’Olmo and Mork, 2014; Dall’Olmo et al., 2016; Organelli et al., 2017) and radiance sensors (Leymarie et al., 2018; Wojtasiewicz et al., 2018), which will enable greater understanding of the biogeochemical structure and possibly additional data for sensor validation. Furthermore, additional sensors could provide observations on biological diversity (Boss et al., 2018).

## OPERATIONAL OCEAN-COLOUR APPLICATIONS

The operational applications of ocean-colour data are described in International Ocean Colour Coordinating Group [IOCCG] (2008) and include *inter alia*: natural fisheries; marine protected area selection and monitoring; ecosystem model data assimilation; aquaculture site selection and monitoring; water quality and eutrophication; hazard monitoring such as nuisance or harmful algal blooms (HAB); dredging/dumping; coastal erosion; or sediment plumes.

This section presents a few examples of ocean colour applications; it is not intended to be exhaustive, and it is recognised that there are very many operational or pre-operational services and applications ongoing or planned. Instead, it is meant to give a flavour of current activities and future developments, with an emphasis on activities in developing countries.

### Harmful Algal Blooms and Aquaculture in South Africa

Aquaculture is a growing industry in South Africa, with the majority of the marine sector focusing on the in-water culture of the mussel *Mytilus galloprovincialis* and the Pacific oyster *Crassostrea gigas*, as well as the land-based farming of the abalone *Haliotis midae* (daff, 2016). All of these industries are to some extent susceptible to the negative impacts of HABs, whether directly through the assimilation of toxins, mechanical damage, or indirectly through anoxia from bloom-collapse (Pitcher and Calder, 2000). As a result, HABs have the potential to cause devastating economic losses to the aquaculture industry.

**FIGURE 5 |:**
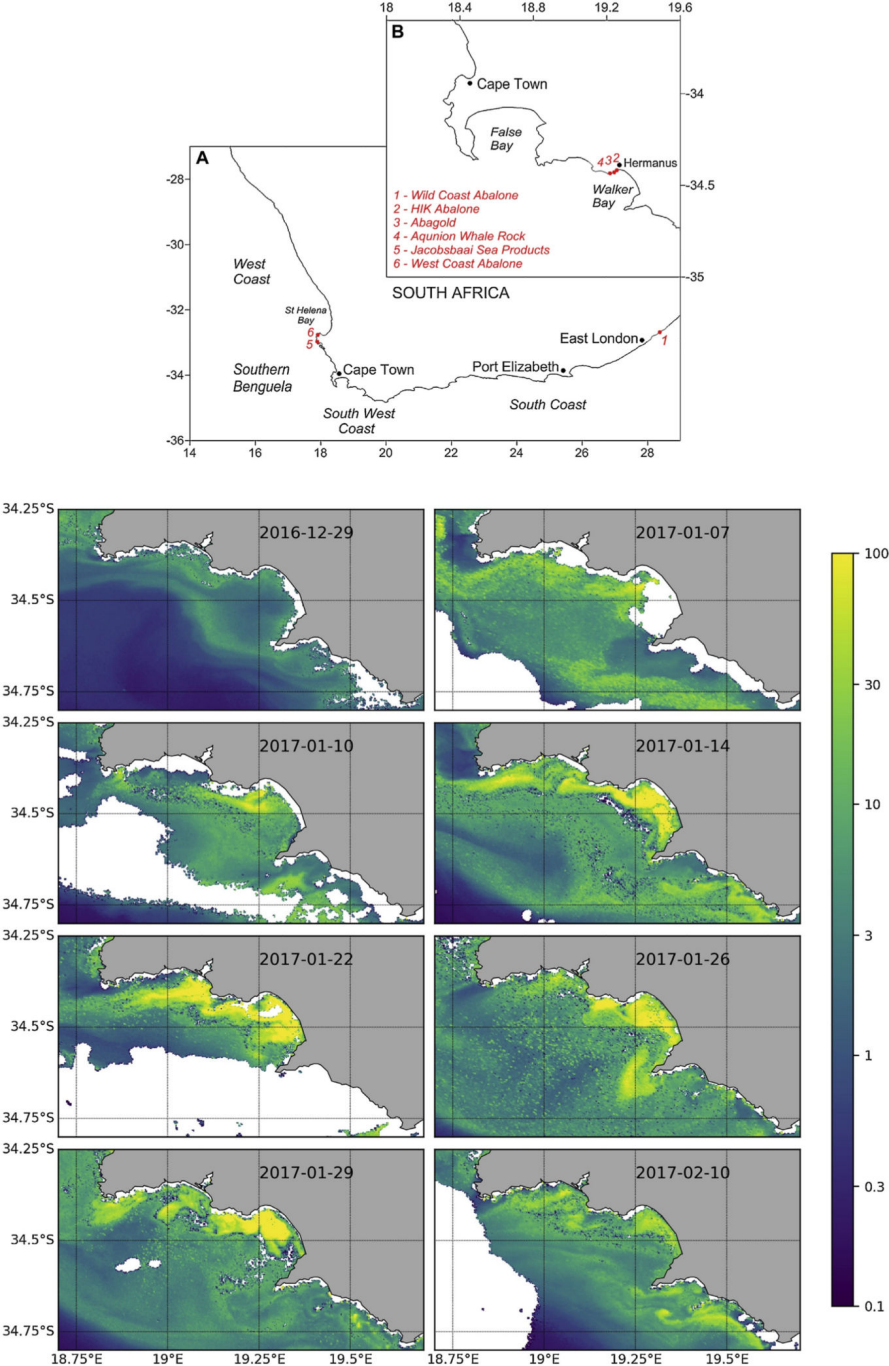
**(A,B)** Map depicting the location of abalone farms on the South African coastline; lower panel shows satellite-derived chl-a concentration (in mg m-3) from the OLCI sensor for the Walker Bay area between the 29th of December 2016 and the 10th of February 2017. White areas represent either cloud or chl-a algorithm failure (Pitcher et al., 2019).

An example of such an event occurred in January 2017 in the vicinity of Walker Bay, Hermanus where a yessotoxin-producing dinoflagellate bloom (Pitcher et al., 2019) led to the mortalities of approximately 250 tonnes of farmed abalone, with an estimated consumer value of $33 million. The spatial and temporal extent of the bloom, captured by S3A-OLCI, is depicted in Figure 5: this used a regional switching algorithm for optimised chl-a estimates in high biomass bloom water types (Smith et al., 2018).

Partially in response to this devastating event, the National Oceans and Coastal Information Management System (OCIMS^32^) of South Africa released an alpha version of a HAB decision support tool in March 2017. While OCIMS supports the general socio-economic potential and ecological conservation of South Africa’s oceans and coasts, the HAB decision support tool specifically provides information to the aquaculture industry and environmental managers, allowing the timely implementation of appropriate mitigation steps.

Looking to the future, operational HAB monitoring services based on EO are likely to significantly expand as the industry develops worldwide, and notably in Africa: aquaculture contributes ~50% to global fish supply, but < 1% of South Africa’s fish supply. The availability of regular satellite-based hyperspectral data may assist in discrimination of certain HAB species (Kurekin et al., 2014) but only if there are distinct spectral features (e.g., as found in *Mesodinium rubrum*).

### Use of Ocean Colour Data for Location of Potential Fishing Zones (PFZ)

**FIGURE 6 |:**
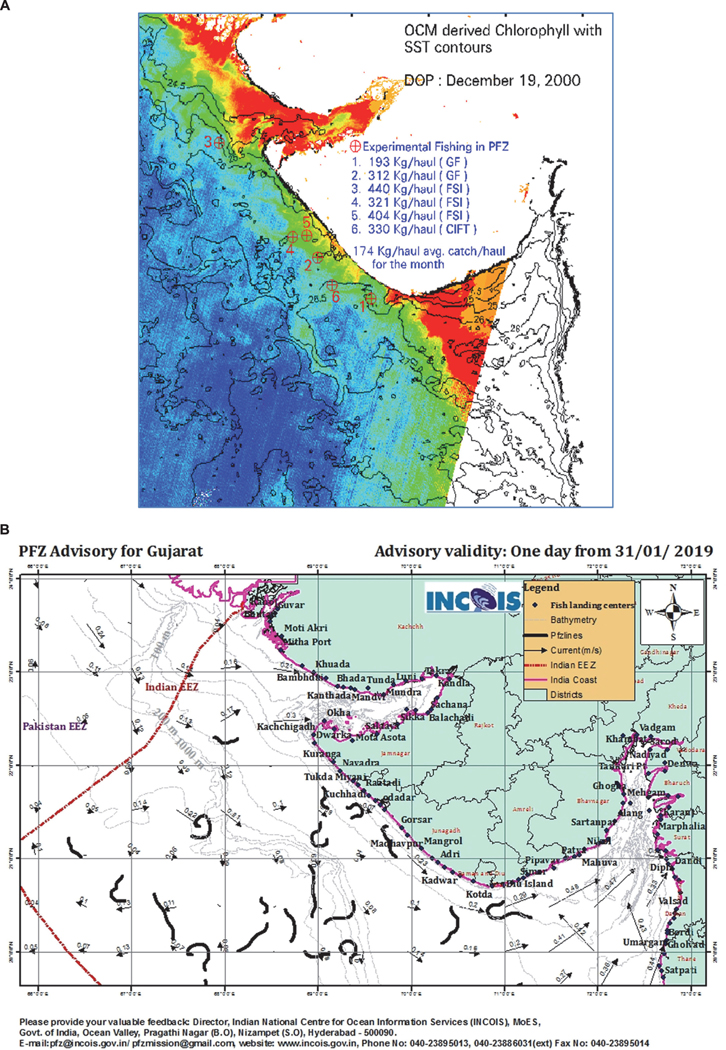
**(A)** Ocean Colour Monitor-derived chl-a concentration with overlaid SST contours used to find PFZ regions. **(B)** Example PFZ advisory issued by INCOIS, Hyderabad on a routine basis for the Orissa coast of India showing both potential fishing zones (noted as ‘Pfzlines’) as well as restricted fishing areas (‘Restricted_zones’). Such advisories are provided for the entire Indian coastline (Credit: INCOIS, Hyderabad; see https://incois.gov.in/MarineFisheries/PfzAdvisory).

Commercial exploitation of natural fishery resources can be made more efficient through use of information on fish location and abundance. Since phytoplankton forms the base of the food chain, ocean colour information indicates productive regions of the marine environment. Complementing ocean colour images of chl-a, sea surface temperature (SST) images provide information on oceanographic processes such as upwelling, convective mixing, etc., important for enrichment of nutrients and increasing productivity. An integrated approach using ocean colour from the Indian OCM sensor and SST from NOAA AVHRR data was developed (Figure 6A) and made operational for potential fishing zone (PFZ) forecasts (Dwivedi et al., 2005). Currently, ocean colour and SST data are used by the Indian National Centre of Ocean Information System (INCOIS) for locating PFZ based on oceanographic features including gradients in SST and colour, fronts, eddies, rings, meanders, and upwelling regions. PFZ advisories (Figure 6B) are generated by INCOIS routinely using OCM-2 data for dissemination to fishers (Chauhan and Raman, 2017) across the entire Indian coast. The bulletins are widely disseminated as maps and text, via fax, phone, newspapers, internet, e-mail, electronic display boards, radio and TV broadcasts, and information kiosks. A cost–benefit analysis to assess the impact of using satellite PFZ forecasts on fish catch showed that the value of the fish catch was higher than the cost of generating PFZ maps and the fishing effort, indicating the value of satellite data in improving the economics of fish catch (Nayak et al., 2003). The bulletins also show restricted zones based on information such as habitats of species including whale sharks off the Gujarat coast during winter monsoon (Kumari and Raman, 2010) or turtle habitats [based on the impact of longline fisheries on turtle bycatch (Bhatpuria et al., 2015)]. Such ‘No Fishing Zones’ can be selected by environmental managers and policy makers to reduce environmental impacts from fishing. Finally, PFZ maps are not produced during periods when fishing is banned, creating an incentive for fishers to observe the ban. The goal of PFZ advisories is to improve catch per fishing effort and not to encourage overfishing.

### Geostationary Sensor Monitoring of a Harmful Algal Bloom

**FIGURE 7 |:**
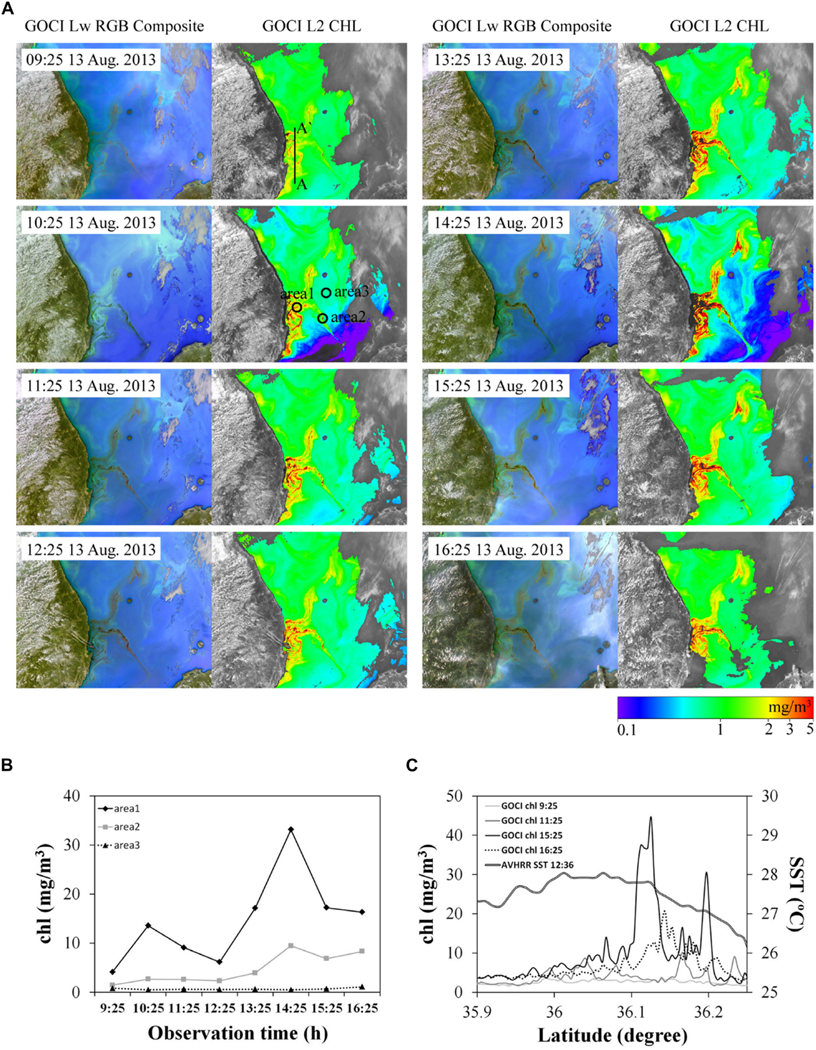
**(A)** Geostationary Ocean Colour Imager radiance composite images and corresponding chl-a images from 09:25 to 16:25 on 13 August 2013: the chl-a profile line (AA′ ) and the hourly chl-a investigation points (1–3) are marked; **(B)** Hourly GOCI chl-a values at points 1–3; and **(C)** chl-a values along line AA′ at 09:25, 11:25, 15:25, and 16:25 local time.

Since 1995 blooms of the harmful dinoflagellate *Cochlodinium polykrikoides* have frequently appeared and caused damage to aquaculture in Korean coastal waters. Choi et al. (2014b) used the Korean GOCI geostationary sensor to monitor diurnal variations in a bloom of *C. polykrikoides* identified through *in situ* measurements. Eight hourly water-leaving radiance composite images and corresponding chl-a images, on August 13, 2013, enabled monitoring of diurnal variations in spatial structure and concentration. HAB species are known to vertically migrate finding optimal conditions, in terms of light, temperature and nutrients (Schofield et al., 2006): cells of *C. polykrikoides* along the Korean coast begin to ascend before sunrise and begin to descend at about 16:00 (Kim et al., 2010). Figures 7A,B clearly show the temporal variations in GOCI-derived chl-a, particularly in the area where *C. polykrikoides* cell abundance is high (area 1 and area 2) with highest chl-a at 14:25. Chl-a profiles extracted from the GOCI data (Figure 7C) show the gradual increase in chl-a until 14:25 in the HAB area. It is worth noting that a sunsynchronous sensor over-passing earlier in the day would detect much lower concentrations in the bloom.

Over the next decade diurnally resolved observations from GEO satellites in far east Asian waters will be possible from Korea’s GOCI-II and China’s HY-3C due for launch in 2019 and 2023, respectively. Although no sensors are planned for other regions, nano-satellite constellations or high-altitude pseudo-satellites may allow similar diurnal observations, albeit regionally. Furthermore, analysis of such diurnal observations should give insights into satellite data merging techniques that composite over 1 day from sensors with differing overpass times.

## ECOSYSTEM MODELLING

Marine ecosystem models are being used increasingly to investigate ecosystem functioning, predict marine changes in response to different climate change scenarios or for supporting ecosystem management in relation to, e.g., eutrophication, HABs and hypoxia (see the reviews by Fennel et al., 2019). Ocean-colour data are being used for assessing the skill of model simulations of chl-a fields (see, e.g., the review by Gregg et al. (2009) or phytoplankton community structure (Hirata et al., 2013), and increasingly to improve simulations by means of data assimilation (see the review by Ford et al., 2018). Data assimilation (DA) algorithms correct simulations toward Earth observations (both satellite and *in situ*), to provide better estimates of the ‘true’ state of the ecosystem, taking account of the uncertainties in the models and in the data. In practice, DA is operated either to estimate parameter values in a model (e.g., Roy et al., 2012; Hoshiba et al., 2018) or to directly adjust the model state-variable simulation with the data (e.g., Tsiaras et al., 2017), or for simultaneous state and parameter estimation (e.g., Gharamti et al., 2017).

**FIGURE 8 |:**
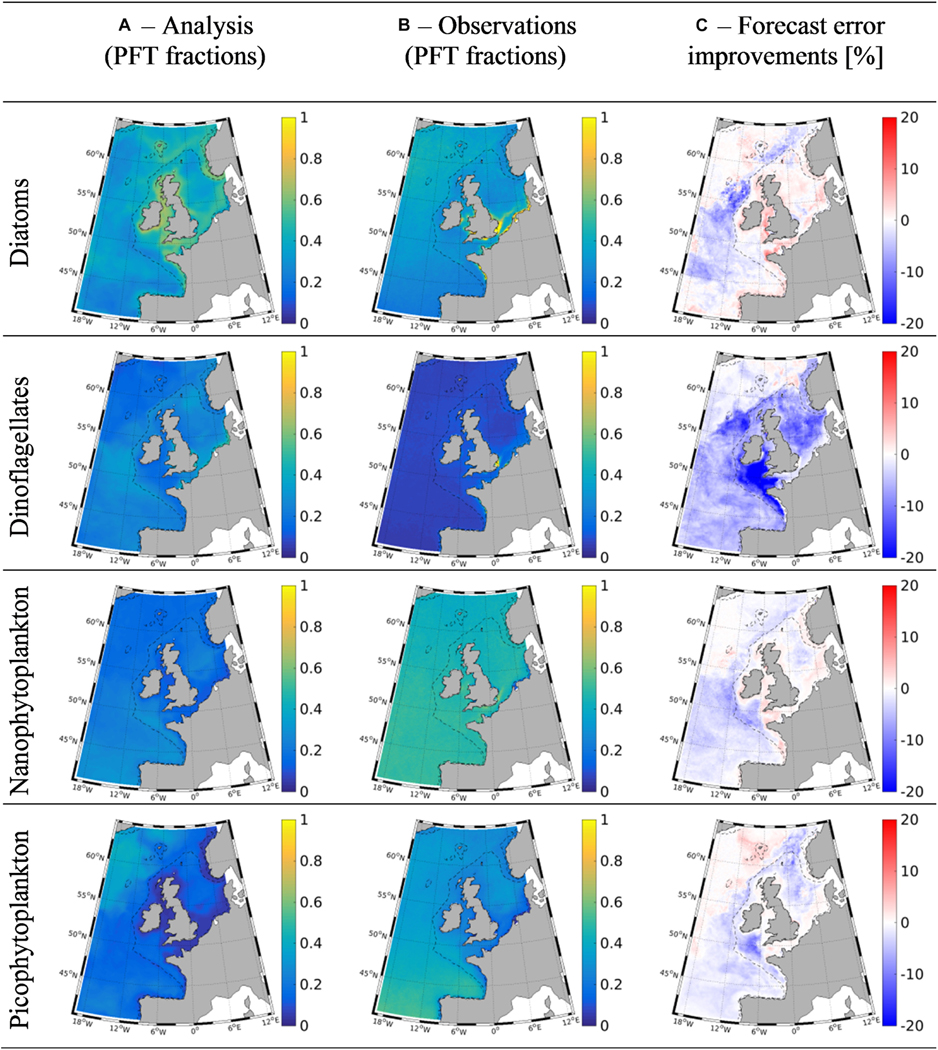
Improved simulation of the plankton community structure by assimilating ocean-colour plankton functional type (PFT) products into a marine ecosystem model of the North East Atlantic. Column **(A)** PFTs fractions from the assimilative simulation (i.e., ratio of the PFT chlorophyll to the total chlorophyll); **(B)** PFTs fractions from ocean-colour data; and **(C)** percentage difference between the RMSD of the PFT assimilation (1-month forecasts) and of the reference model simulation without assimilation; RMSD is the root-mean-square-deviation between grid-point time series of model output and ocean-colour data. Figure elaborated from Figure 8 in Ciavatta et al. (2018), delivered by the Copernicus Marine Environment Monitoring Service TOSCA project.

Data assimilation of ocean colour is, arguably, not as widespread as assimilation of physical variables: for example, in the European Copernicus Marine Environment Monitoring Service, ocean-colour assimilation is run for operational reanalysis and forecasts in the Mediterranean Sea (Teruzzi et al., 2018) and for operational reanalysis in the North West European Shelf, while the implementation for short-term forecasting is ongoing (Skakala et al., 2018). The seminal work using CZCS ocean-colour for data assimilation involved ‘nudging’ a simple model to the chl-a observed value to evaluate ocean productivity (Armstrong et al., 1995). The most recent applications use an expanded suite of ocean-colour products with multivariate DA techniques that can correct the whole set of ocean variables in complex, highly non-linear models. For example, the chl-a time series from ESA’s CCI Ocean Colour (see section ‘Time Series for Climate’) has been assimilated in applications ranging from mapping the risk of oxygen deficiency in shelf-bottom waters (Ciavatta et al., 2016) to improving global estimates of carbon cycle variables (Ford and Barciela, 2017). DA of new ocean-colour products, such as phytoplankton functional types (PFTs), outperformed total chlorophyll DA in improving the reanalysis of the plankton community structure (Figure 8; Ciavatta et al., 2018) and the pre-operational prediction of some biogeochemical indicators (e.g., CO2 fugacity, Skakala et al., 2018).

Moving forward, the development of bio-optical modules in marine ecosystem models is opening new frontiers for ocean-colour DA (see e.g., the model by Dutkiewicz et al., 2015). Assimilation of ocean-colour phytoplankton absorption (Shulman et al., 2013), spectral diffuse attenuation coefficient (Ciavatta et al., 2014), remote sensing reflectance (Jones et al., 2016), absorption of CDOM and detritus (Gregg and Rousseaux, 2017) have been shown to enhance the estimation of the bio-optically active compounds, including in case II waters. This approach is tackling the issue of possible mismatches between the variables that are simulated by models and those retrieved from ocean colour (e.g., observed ‘chlorophyll’ concentration might include simulated suspended solids in coastal areas, Jones et al., 2016). Finally, simulating optical properties can allow one to use ecosystem models for design and analysis of future satellite missions, e.g., for the selection of bands in hyper-spectral remote sensors (Gregg and Rousseaux, 2017).

## TRAINING AND CAPACITY BUILDING

Capacity building and training, notably in developing countries, is recognised as an on-going need to make best use of the ocean colour resources and data sets available to the user community. The IOCCG has supported regular lecture series at the Laboratoire d’Océanographie de Villefranche (LOV) in Villefranche-sur-Mer: the lectures and other materials are available on the IOCCG web site, and in the past, has supported training courses in various locations around the world. EUMETSAT undertakes marine training courses in Europe and around the world. The Partnership for Observation of the Global Ocean (POGO) offers a number of training, education and fellowship opportunities. Below are described some ocean-colour relevant initiatives contributing to community and capacity building and training.

### Group on Earth Observations EO4SDG

EO4SDG is a GEO initiative on promoting EO for Sustainable Development Goals^33^. It has three goals ‘(1) Demonstrate how Earth observations, geospatial information, and socio-economic and other data contribute in novel and practical ways to support achievement of the SDGs. (2) Increase skills and capabilities in uses of Earth observations for SDG activities and their broader benefits. (3) Broaden interest and awareness of Earth observations support to the SDGs and contributions to social, environmental, and economic benefits’ (EO4SDG Implementation Plan: GEO Initiative 18: Earth Observations in Service of the 2030 Agenda for Sustainable Development, available at eo4sdg.org).

### The Chlorophyll Globally Integrated Network (ChloroGIN)

ChloroGIN^34^ (Sathyendranath et al., 2010) was an early attempt to create a network of regional ocean-colour data providers and users. Mapped level 2 satellite ocean-colour products were made available to the users through a single portal. The Africa data production has been maintained with complete coverage of African waters at 1-km since 2010. Data have been used in a number of capacity building projects in Africa such as the Monitoring for Environment and Security in Africa (MESA) with data available via the web site and also via Eumetcast. The data continue to be used in the follow-up GMES and Africa project and production is transitioning to Sentinel 3 OLCI. ChloroGIN has had a strong capacity building goal, and though there has been little resource of late for global coordination of this activity, the regional nodes within ChloroGIN have remained active, and become more self-sustained (see for example, the case of GMES Africa and Antares, the Latin American networks detailed below). But the benefits from enhanced global coordination have not yet been fully realised, because of lack of funding.

### ANTARES Latin and South American Network

The Antares network was created in July 2003 under the auspices of the IOCCG, Partnership for Observation of the Global Ocean (POGO), Nippon Foundation and Inter-American Institute for Global Change Research (IAI). Antares served as a seed for ChloroGIN. Antares aims to monitor and detect long-term changes in coastal waters around Latin-America with the main approach of developing time-series of oceanographic observations (physical, chemical and biological) integrated with remote sensing data, products and modelling. Capacity building as well as scientific and technical collaboration are key for Antares network. Current participating countries are Argentina, Brazil, Chile, Colombia, Ecuador, Mexico, Peru, United States, and Venezuela.

The Antares stations are located in a region with scarce *in situ* observations. They are distributed along the Pacific, Caribbean and Atlantic coasts of Latin America, covering about 70 degrees of latitude from 31° N (Ensenada, Mexico) up to 38° S (EPEA, Argentina). The time-series observations carried out at these sites register valuable ecological information in different areas with different oceanographic characteristics. Among the Pacific stations, one is located in the northern hemisphere (Ensenada, Mexico) and another in the southern hemisphere (IMARPE, Peru). The Caribbean station (CARIACO, Venezuela) is no longer operational and was located on a basin more than 1000 m deep. On the South Atlantic side, Ubatuba (Brazil) located in a Subtropical regime and EPEA (Argentina) on a temperate regime. Data will be made available via the Inter-American Institute for Global Change Research (IAI) Open Data Portal^35^.

Through an IAI project, Antares is studying trends in phytoplankton and its associated ecosystem services in a context of climate variability. It aims at making more ‘visible’ the link between phytoplankton and the concentration of CO_2_, a key variable for the biogeochemistry of the planet and the organisation of human society.

Satellite ocean colour imagery is available via the Sigma-Antares tool, at antares2.cptec.inpe.br for the entire coast of Latin and South America.

### ESA Earth Observation for Sustainable Development

ESA’s EO4SD^36^ is a major project promoting use and uptake of EO data (e.g., Anderson et al., 2017) to support the UN Sustainable Development Goals by working with International Funding Institutes like the World Bank or East Asian Development Bank and their individual client states. The marine and coastal project has just started (mid 2018) and is one of eight including, *inter alia*, water resource management, disaster risk reduction and climate resilience. The marine and coastal project aims at supporting a number of services, including water quality monitoring, aquaculture site selection, invasive species monitoring together with near coastal and high-resolution services, coastal and benthic habitat status and MPA management. The project aims to address five regions worldwide: west Africa, western Indian Ocean, Caribbean, east Asia and Pacific Islands.

### Global Monitoring for Environment and Security and Africa (GMES and Africa)

Global Monitoring for Environment and Security and Africa is a pan-continental project using EO data, technologies and services in support of African sustainable development; it is, arguably, an exemplar of construction of an integrated ocean and coastal motoring system in developing countries. As a joint initiative between the African and European Unions, there is strong emphasis on realising value from the data, information, expertise and know-how offered by the Copernicus programme. In the marine and coastal domains, four consortia around the continent are developing and implementing a variety of services, including the provision of oceanographic products, fisheries and aquaculture support, and coastal ecosystem monitoring and assessment. Private companies are also encouraged to participate in the services with 20% of the regional budget reserved for the sector. From an ocean-colour perspective, Sentinel 2 and Sentinel 3 in particular will provide substantial value to these services, using a variety of products and approaches.

In South and East Africa under the ‘MarCoSouth’ consortium, application services will provide additional value to ocean colour science products, with user co-design a major aspect of the service development. Standard Copernicus reduced resolution (1-km) Sentinel 3A and 3B OLCI products will provide base ocean biogeochemistry products for the African shelf. In addition to standard chl-a and Total Suspended Matter products, regional switching algorithms will be used for optimised chl-a estimates in high biomass bloom water types. OLCI Full Resolution data at 300 m resolution, and Sentinel 2A/SB MSI derived products will be provided for specific coastal sites, prioritised for aquaculture, desalination, estuarine conservation and fisheries, effluent discharge and other applications. Three application services will provide additional value to these ocean colour science products:

- Aquaculture support will focus primarily on determining HAB risk, gross water quality monitoring and temperature range analysis for a range of target organisms, both in near-real time and for historical analysis. A range of sectors will be targeted, including the relatively high-technology and high-investment abalone farms in South Africa (see section ‘Harmful Algal Blooms and Aquaculture in South Africa’), bivalve operations in Namibia and South Africa, industrial and artisanal finfish farming regionally, and artisanal seaweed farming in Tanzania and Kenya.
- Fisheries support is already well established for coastal fisheries in West Africa and large pelagics in the Western Indian ocean. In Southern Africa, new services will focus on the provision of ocean colour and SST frontal maps – primarily for pelagic fisheries such as sardine and tuna – across the shelf and in Lake Victoria. PFZ products will also be provided through mobile platforms (see section ‘Ground Segment and Data Processing’). Longer term development will focus on integration of catch data, refinement of PFZ products through chl-a/SST heat maps, and provision of longer-term phenologies and time series analyses to national and regional resource management agencies.
- The ecosystem monitoring service will focus primarily on reef and mangrove mapping and assessment from a habitat perspective, with additional risk monitoring capabilities at prioritised sites for high-resolution water quality and Virtual Buoy oceanographic monitoring. Routine near-real time products from Sentinel 2 and 3 will play a major role here, monitoring for effluent discharge and turbidity, eutrophication and temperature anomalies.

### Partnership for Observation of the Global Ocean (POGO)

Partnership for Observation of the Global Ocean^37^ offers various ocean-colour relevant training education and fellowship opportunities including, *inter alia*, the Nippon Foundation (NF)-POGO sponsored Centre of Excellence for training in observational oceanography hosted currently at the Alfred Wegener Institute, Germany and regional training courses; a fellowship training programme in collaboration with the Scientific Committee on Oceanic Research (SCOR) to support young professionals from developing countries to spend up to 3 months at a major oceanographic institution; and shipboard training.

## OCEAN COLOUR WITHIN OCEAN OBSERVING SYSTEMS

Developments since the last OceanObs conference in 2009 have rapidly expanded the applications that make use of satellite ocean-colour data. The record has extended to 22 years (in 2019); a virtual constellation of operational missions is now in orbit, with replacements planned; routine global observations are available at 300 m; production of ocean-colour observations has developed significantly with operational production of L1, L2 and L3 data by Copernicus in Europe, and NOAA and NASA in the United States (for example); and access to data is now possible through a variety of methods including ftp, OPeNDAP and web-GIS, while cloud platforms offer greater flexibility for data analysis (see section ‘Ground Segment and Data Processing’). Areas still to be addressed include higher spatial resolution, for observation of near-coastal or inland waters, and diurnal observations through the day (which are indispensable for the meteorological community) with geostationary missions and only east Asia has such a mission in place. Since this paper is associated with OceanObs 2019 it is instructive to consider how ocean colour will contribute to the aims of the conference to ‘improve response to scientific and societal needs of a fit-for-purpose integrated ocean observing system, for better understanding the environment of the Earth, monitoring climate, and informing adaptation strategies as well as the sustainable use of ocean resources^38^.’

Ocean colour is central to ocean observing systems, providing the only direct global observations of phytoplankton, the base of the ocean ecosystem. Such observing systems are driven by the United Nations Sustainable Development Goals (SDGs) and the Convention on Biological Diversity (CBD). Ocean colour is noted as a cross-cutting Essential Ocean Variable (EOV) by GOOS, is implicated in other EOVs and is relevant to a number of GEO-BON Essential Biodiversity Variables (Muller-Karger et al., 2018). Observing systems operate at a number of spatial and temporal scales reflecting the range of applications from climate observation (global, long-term) to operational (usually regional or local, short-term); nevertheless, some commonalities can be drawn.

Ocean colour is fundamental to observing the ocean ecosystem and carbon system including primary production, particulate organic or inorganic carbon and dissolved organic carbon; these also require inputs from multiple disciplines such as sea-surface temperature or solar irradiance, encouraging collaboration between these communities. Extending the primarily surface observations to the ocean interior will need synergy with *in situ* data, notably from BGC-Argo floats in oceans, instrumented gliders or moorings in shelf-seas and novel *in situ* plankton monitoring systems (Lombard et al., 2019). Likewise, stepping from observations to forecasts implies data assimilation into models or validation of models using the ocean-colour record (see section ‘Ecosystem Modeling’ and Fennel et al., 2019). All these considerations lead to the need to sustain ocean-colour observations in operational mode; further improve ocean-colour observations and the derived products (including data uncertainty characteristics) by improving the constituent retrieval algorithms, through strict adherence to international protocols and thorough uncertainty characterisation of instrumentation for *in situ* data gathering (see section ‘Ocean Colour EO Sensors and in situ Observations’); improve the methods to remove atmospheric contamination and combine multiple sensors (e.g., see section ‘Time Series for Climate’); and improve access to ocean-colour data, following international standards and conventions for data storage (like netCDF) or data serving (like OGC compliant web servers). There is also an obvious need for greater training opportunities for specialists and non-specialists alike, in both developed countries and developing countries, to make use of the wider range of products, where issues of impact of climate change, of food security, of water availability and biodiversity loss are particularly acute.

## CONCLUSION AND FORWARD LOOK

- The conclusions in this section focus on ocean, shelf and off-shore coastal applications: the reader is reminded that near-coastal, estuarine and inland water observations are not considered herein.
- Operational satellite sensor constellations, such as Copernicus Sentinel 3 OLCI and NOAA JPSS VIIRS, will ensure provision of ocean-colour data as elements of integrated, sustained observing systems. This ensures that commercial and governmental operators who invest in ocean-colour-related services can do so with the confidence that continuity of data is guaranteed. The operational missions will be complemented by new capability missions such as NASA PACE.
- Over the next decade the contiguous ocean-colour record will cover 30 years, reaching the minimum length of time series essential for discrimination of climate change impacts from shorter-scale natural variability, at least for some regions.
- The higher spatial resolution available from Sentinel 3 OLCI, 300 m globally with full ocean colour capability, will support applications closer to the coast as well as in estuaries and inland waters.
- Although not considered explicitly herein there is a gap in high-resolution (~ < 10 m) observations of ocean colour (or inland water colour) and it is recommended that space agencies consider this as a future development. Sentinel 2 MSI and Landsat 8, although not optimised for aquatic applications, can provide observations of features such as sediment plumes, floating vegetation and phytoplankton information in some cases, at the 10–100 m resolution required for coastal and fresh-water systems.
- Significant steps are being taken to provide analysis-ready data in a variety of formats, with increasingly faster turnaround from data capture to product delivery to the enduser, which will support applications that need a rapid response such as alerts for nuisance or harmful algal bloom in the vicinity of aquaculture farms and beaches.
- There is a continuing need to improve retrieval algorithms, as well as the methods to establish the quality of their performance: hence, it is recommended that strict protocols for *in situ* data gathering be followed, with full system uncertainty characterisation, to obtain Fiducial Reference Measurements. This will help ensure that combined datasets used for algorithm construction and satellite product validation and product evaluation have minimal inter-investigator differences.
- As hyperspectral satellite sensors are about to become a reality, it is important to explore novel algorithms to reduce uncertainties in products by exploiting the new capability.
- Continuity in campaigns to obtain Fiducial Reference Measurements supporting system vicarious calibration and validation of sensors is key to the success of ocean-colour missions, both when they join the ocean-colour constellation and as they extend the duration of their operation in space. This is an area that needs to be funded continuously by space agencies.
- Novel instrumentation, including autonomous or flow-through systems installed on research vessels or ships of opportunity, have the potential to greatly increase the number of satellite ‘matchup’ data but they need to follow strict protocols with respect to appropriate system characterisation, calibration, and maintenance including treatment of bio-fouling. Such systems will also help characterise the variability within a satellite pixel and hence reduce the retrieval uncertainties. Furthermore, additional sensors could provide observations on biological diversity (Boss et al., 2018).
- EO data will be increasingly combined with operational *in situ* sensors to provide integrated observations with the temporal or depth capability of *in situ* data complementing the synoptic spatial scale of satellite EO data.
- Given the value and cost of *in situ* data it is important that international or de facto standards are followed that allow linking or interoperability when data are held in multiple databases.
- Ocean colour data will be increasingly used for initialisation and updating of short-term numerical forecast models as well as validating hindcast climate model runs.
- Mobile communication is expected to grow worldwide, especially in developing countries, supporting citizen science and communication of EO-based information to citizens: this two-way communication will both aid the ocean-colour field and increase scientific awareness.
- Capacity building and training will remain important to fully utilise satellite observation and integrated observing systems for societal benefit worldwide, but notably in developing countries.
- Finally, the UN Sustainable Development Goals aiming for the implementation of the 2030 Agenda for Sustainable Development will set a wide context for developments in the ocean colour field for the next decade.

## Figures and Tables

**TABLE 1 |:** The Global Climate Observing System uncertainty requirements for the ocean colour ECV

Products	Frequency	Spatial resolution	Required measurement uncertainty	Stability/decade
Water-leaving radiance	Daily	4 km	5% in blue and green wavelengths	0.5%
Chl-a concentration	Weekly averages	4 km	30%	3%

**TABLE 2 |:** Recent, existing, and near-future satellite sensor systems of relevance for ocean colour (revised and updated after International Ocean Colour Coordinating Group [IOCCG], 2018).

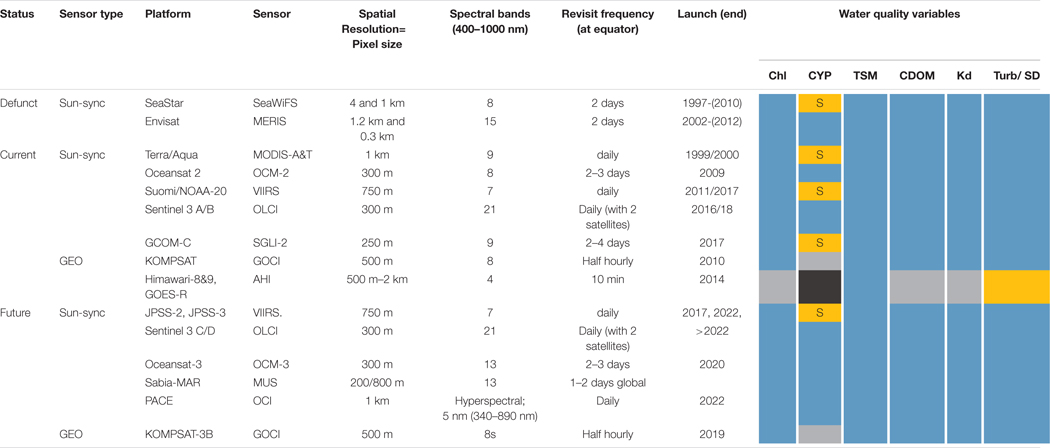

■: highly suitable, ■: suitable, ■: potential, ■: not suitable, Chi, Chlorophyll; CYP, cyanobacteriaI pigments (S denotes surface blooms); TSM, total suspended matter; CDOM, coloured dissolved organic matter; Kd, diffuse attenuation coefficient (or attenuation coefficient of diffuse light); Turb, Turbidity / SD, Secchi Disk Depth.

**TABLE 3 |:** Recent, existing, and near-future satellite sensor systems of relevance for inland and near-coastal water quality (revised and updated after International Ocean Colour Coordinating Group [IOCCG], 2018).

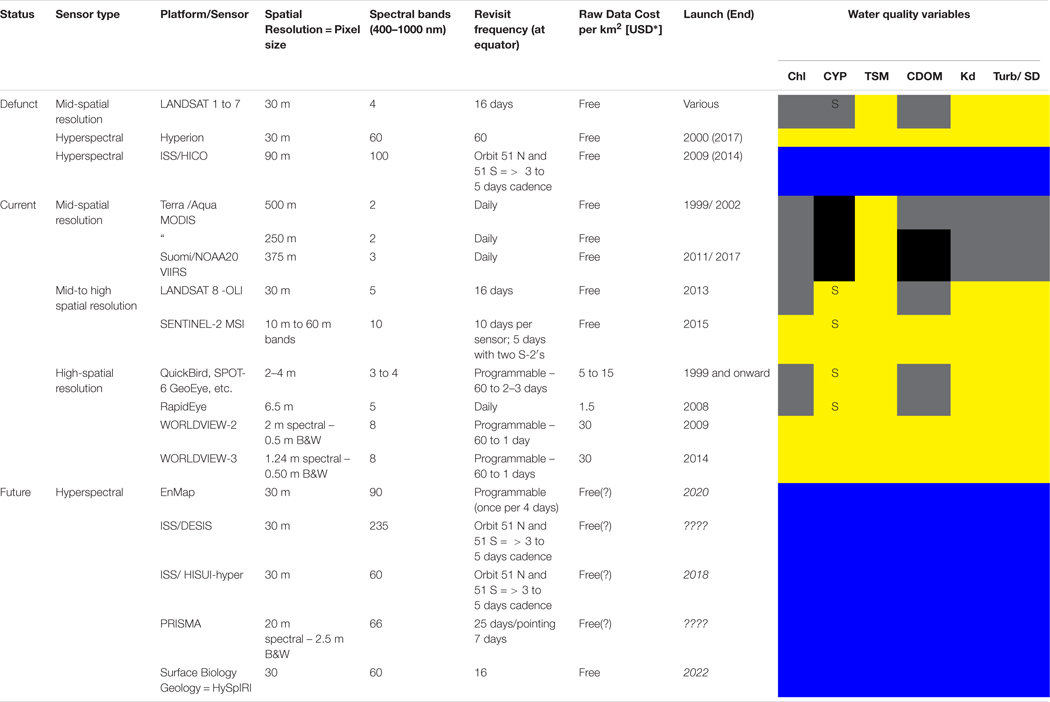

■: highly suitable, ■: suitable, ■: potential, ■: not suitable. CHL, Chlorophyll; CYP, cyanobacteriaI pigments; TSM, total suspended matter; CDOM, coloured dissolved organic matter; Kd, diffuse attenuation coefficient (or attenuation coefficient of diffuse light); Turb, Turbidity; SD, Secchi Disk Depth. Free, publicly available; Free(9), available upon request for research.

## Footnotes

1
www.ioccg.org

2
http://ceos.org/ourwork/virtual-constellations/ocr/

3
https://www.ncdc.noaa.gov/gosic/gcos-essential-climate-variable-ecv-data-access-matrix/gcos-ocean-biogeochemistry-ecv-ocean-color

4
http://www.esa-oceancolour-cci.org/?q?webfm_send/582

5
https://geoblueplanet.org/

6
http://www.goosocean.org

7
https://geobon.org/ebvs/what-are-ebvs/

8
https://www.un.org/en/events/biodiversityday/convention.shtml

9
http://www.undp.org/content/undp/en/home/sustainable-development-goals.html

10
https://www.jpss.noaa.gov

11
https://pace.oceansciences.org/docs/PACE_polarimetry_final.pdf

12
https://uncw.edu/socon/mission.html

13
http://ioccg.org/wp-content/uploads/2016/02/INSITU-OCR-white-paper.pdf

14
http://www.hypernets.eu/from_cms/objectives

15
https://www.pangaea.de

16
http://biogeochemical-argo.org/

17
http://www.secchidisk.org/

18
http://www.eyeonwater.org/

19
https://cyanos.org/

20
http://step.esa.int/main/download/

21
https://oceancolor.gsfc.nasa.gov/

22
https://www.star.nesdis.noaa.gov/sod/mecb/color/

23
https://coastwatch.noaa.gov/

24
www.oceancolour.org

25
http://moi.govmu.org/mesa/

26
https://data.worldbank.org/indicator/IT.CEL.SETS

27
www.statista.com

28
http://abalobi.info/

29
www.oceancolour.org/portal

30
https://giovanni.gsfc.nasa.gov/giovanni/

31
https://www.esa.int/Our_Activities/Observing_the_Earth/Copernicus/

32
https://eo.meraka.csir.co.za/hab/app/

33
http://eo4sdg.org

34
www.chlorogin.org

35
http://www.iai.int/en/post/detail/open-dataopen-science

36
http://eo4sd.esa.int/

37
www.ocean-partners.org/

38
http://www.oceanobs19.net/
